# Novel 11β-hydroxysteroid dehydrogenase 1 inhibitors reduce cortisol levels in keratinocytes and improve dermal collagen content in human *ex vivo* skin after exposure to cortisone and UV

**DOI:** 10.1371/journal.pone.0171079

**Published:** 2017-02-02

**Authors:** Stéphanie M. Boudon, Anna Vuorinen, Piero Geotti-Bianchini, Eliane Wandeler, Denise V. Kratschmar, Marc Heidl, Remo Campiche, Eileen Jackson, Alex Odermatt

**Affiliations:** 1 DSM Nutritional Products Ltd., Kaiseraugst, Switzerland; 2 Division of Molecular and Systems Toxicology, Department of Pharmaceutical Sciences, University of Basel, Basel, Switzerland; University of Alabama at Birmingham, UNITED STATES

## Abstract

Activity and selectivity assessment of new bi-aryl amide 11β-hydroxysteroid dehydrogenase 1 (11β-HSD1) inhibitors, prepared in a modular manner via Suzuki cross-coupling, are described. Several compounds inhibiting 11β-HSD1 at nanomolar concentrations were identified. Compounds **2b**, **3e**, **7b** and **12e** were shown to selectively inhibit 11β-HSD1 over 11β-HSD2, 17β-HSD1 and 17β-HSD2. These inhibitors also potently inhibited 11β-HSD1 activity in intact HEK-293 cells expressing the recombinant enzyme and in intact primary human keratinocytes expressing endogenous 11β-HSD1. Moreover, compounds **2b**, **3e** and **12e** were tested for their activity in human skin biopsies. They were able to prevent, at least in part, both the cortisone- and the UV-mediated decreases in collagen content. Thus, inhibition of 11β-HSD1 by these compounds can be further investigated to delay or prevent UV-mediated skin damage and skin aging.

## Introduction

With an aging population, UV-mediated skin damage and skin aging-related diseases represent an increasing problem and there is an increasing demand for novel therapies against skin diseases [1]. Excessive exposure to UV light results in skin damage, with erythema and DNA damage, oxidative stress, and an inflammatory response with the production of pro-inflammatory mediators such as tumor necrosis factor α (TNFα), interleukin 6 (IL6) and interleukin 1β (IL1β), and the activation of nuclear factor-κB (NF-κB) [2–4]. Glucocorticoids play an important immune modulatory role and by activating glucocorticoid receptors (GR) they suppress the expression of pro-inflammatory cytokines and activation of NF-κB, thereby aiding in the resolution of the inflammatory response [5].

Human skin has the capacity to produce glucocorticoids, androgens and estrogens from *de novo* synthesis of cholesterol via the steroidogenic pathway [6–10]. Besides, the local concentration of cortisol is controlled by 11β-hydroxysteroid dehydrogenase (11β-HSD) enzymes, catalyzing the interconversion of active cortisol and inactive cortisone [11]. 11β-HSD1 is a bidirectional enzyme utilizing cofactor NADPH and acts *in vivo* predominantly as an oxo-reductase converting cortisone to cortisol [12]. It is widely expressed; in skin it has been detected in keratinocytes, dermal fibroblasts and the outer root sheath of hair follicles [13]. In contrast, 11β-HSD2 utilizes cofactor NAD^+^, oxidizes cortisol to cortisone, and is expressed in mineralocorticoid target tissues such as kidney, colon and salivary gland but also in placenta [11], and it has also been found in keratinocytes [14, 15].

The production of glucocorticoids in skin has been shown to be strongly influenced by ultraviolet (UV) radiation. On one hand it has been shown that UVB results in an activation of a dermal regulatory system analogous to that of the hypothalamus-pituitary-adrenal (HPA) axis and stimulation of steroidogenic *de novo* synthesis of cortisol [8, 16, 17], and on the other hand UVB and UVC (but not UVA) exposure led to an enhanced expression of 11β-HSD1 but had no effect on 11β-HSD2 (which was increased by UVA) [14]. These observations indicate that UVB exposure results in increased dermal cortisol production.

Due to their potent effects on the regulation of immune responses, synthetic glucocorticoids are widely used to treat acute and chronic inflammatory diseases [18, 19]. In this respect, topical application of glucocorticoids represent the main treatment option for inflammatory dermatitis, aiming to reduce the infiltration of the skin by inflammatory cells and suppressing inflammatory effects on keratinocytes [20]. Nevertheless, both prolonged systemic and topical treatment with glucocorticoids are known to cause skin atrophy, due to effects on collagen synthesis and degradation and by impacting on keratinocyte and fibroblast proliferation [21–28].

Similarly, chronically elevated 11β-HSD1 activity in aging skin may contribute to glucocorticoid-induced dermal and epidermal thinning and dermal-epidermal junction flattening, reduced dermal fibroblast proliferation and impairment of collagen content [29–32]. Studies in mice deficient in 11β-HSD1 showed higher collagen density, better structured collagen organization and delayed age-induced dermal atrophy compared with age-matched wild-type mice [32]. Additionally, treatment with a selective 11β-HSD1 inhibitor enhanced dermal thickness and collagen content in mice, an effect suggested being a result of a higher number of dermal fibroblasts [31]. Inhibition of 11β-HSD1 by topical and subcutaneous applications of a selective compound increased the number of keratinocytes and dermal fibroblasts in mice [31, 33]. Based on the above mentioned studies it was suggested that pharmacological inhibition of 11β-HSD1 may reverse the decreased collagen content observed in intrinsically and extrinsically aged skin and in glucocorticoid-induced skin atrophy.

Several efforts have been devoted by both academic groups and the pharmaceutical industry towards the discovery of selective 11β-HSD1 inhibitors [34]. The first selective 11β-HSD1 inhibitors, reported by Barf *et al*. [35], were 2-aminothiazoles. These have been followed by different structural classes, and several clinical candidates for the treatment of diabetes and metabolic syndrome have been reported [34, 36]. In the present study, a series of novel biaryl amide compounds was profiled for inhibitory activity against 11β-HSD1, selectivity over 11β-HSD2, 17β-HSD1, and 17β-HSD2, as well as activity in intact cells and *ex vivo* in human skin samples.

## Materials and methods

### Materials and methods for chemistry

Dichloromethane for amidation reactions was dried over sodium sulfate and diethyl ether was dried over phosphoric anhydride, followed by storage under argon. All other reagents were reagent or analytical grade and used as received (for a detailed description of the methods and compounds see S1 File). All air- and water- sensitive reactions were performed under argon. Water for cross-coupling reactions was degassed by sparging with argon under vacuum for 30 min prior to use. The catalytic 10 mM Pd(EDTA) solution was prepared from palladium(II) chloride, ethylenediaminetetraacetic acid (EDTA) disodium salt dihydrate and sodium carbonate as described [37].

### General synthesis strategies

**Fig 1:**
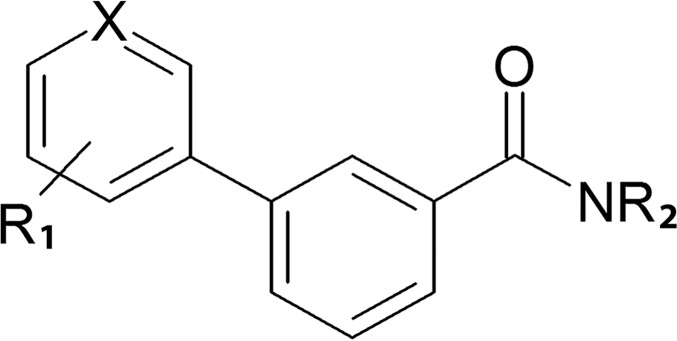
General structure of bi-aryl amide compounds.

**Fig 2:**
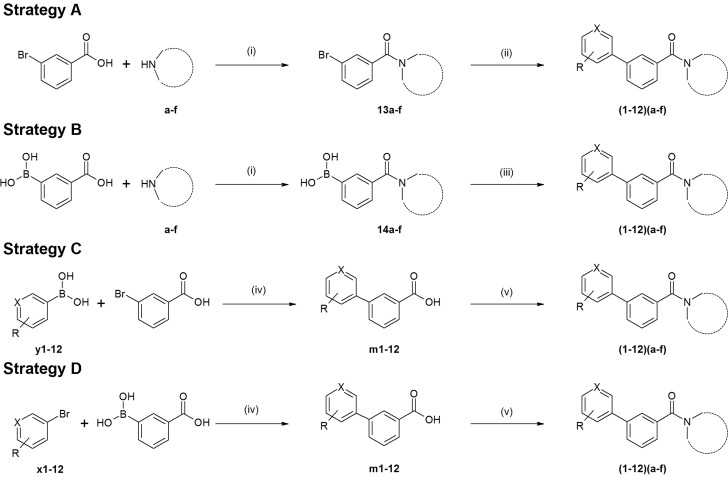
**The four strategies used for the synthesis of the library compounds (1–12)(a-f).** Regents and reaction conditions: (i) *N*-(3-dimethylamino)-propyl-*N’*-ethyl-carbodiimide hydrochloride (EDC×HCl), 1-hydroxybenzotriazole (HOBt), *N*-ethyl-di*iso*propylamine (DIEA); (ii) arylboronic acid **y1-12**, base, palladium catalyst; (iii) aryl bromide **x1-12**, base, palladium catalyst; (iv) base, palladium catalyst; (v) secondary amine **a-f**, EDC×HCl, HOBt, DIEA. Bases and catalysts used in the Suzuki-Miyaura cross-coupling steps are detailed in the Materials and Methods section.

Where not otherwise stated, the library compounds (see Fig 1, S1 Fig, S1 File) were prepared by means of one of the following two-step synthesis strategies (Fig 2).

#### General strategy A

**Step A1.** In a round-bottomed flask 3-bromobenzoic acid was dissolved in anhydrous dichloromethane (5 mL/mmol) under stirring at room temperature and 1-hydroxybenzotriazole (HOBt, 1.11 eq) and *N*-(3-dimethylamino)-propyl-*N’*-ethyl-carbodiimide hydrochloride (EDC×HCl, 1.10 eq) were added. After quantitative activation (as judged by UHPLC analysis), the required secondary amine **a-f** (1.2 eq) and *N*-ethyl-di*iso*propylamine (DIEA, 1.5 eq) were added. After 30 min the mixture was concentrated under reduced pressure, taken-up with ethyl acetate (AcOEt) (40 mL/mmol 3-bromobenzoic acid), washed with 5% KHSO_4_ (2×15 mL/mmol 3-bromobenzoic acid), H_2_O (12 mL/mmol 3-bromobenzoic acid), 5% NaHCO_3_ (3×12 mL/mmol 3-bromobenzoic acid) and brine (12 mL/mmol 3-bromobenzoic acid), dried over Na_2_SO_4_, filtered and evaporated to dryness under reduced pressure to yield aryl bromide intermediate **13a-f**.

**Step A2.** The aryl bromide derivative **13a-f** obtained in the previous step, aryl boronic acid **y1-12** (1.1 eq), K_2_CO_3_ (3 eq) and palladium(0) tetrakis(triphenylphosphine) (0.02 eq) were given in this order in a screw-cap reactor, a 8:8:1 toluene/EtOH/H_2_O mixture (8.5 mL/mmol **13a-f**) was added, the reactor was closed tightly and heated to 100°C under stirring. After 4 h the mixture was cooled to room temperature, diluted with H_2_O (12 mL/mmol **13a-f**), extracted with AcOEt (2×25 mL/mmol **13a-f**), the pooled organic phases were washed with 5% NaHCO_3_ (2×12 mL/mmol **13a-f**) and brine (12 mL/mmol **13a-f**), dried over Na_2_SO_4_, filtered and evaporated to dryness under reduced pressure. If necessary, the crude product **(1–12)(a-f)** was purified by preparative HPLC.

#### General strategy B

**Step B1.** To a suspension of 3-carboxyphenylboronic acid in 3:2 dichloromethane/acetonitrile (anhydrous, 5 mL/mmol) HOBt (1.11 eq) and EDC×HCl (1.10 eq) were added. After complete dissolution the required secondary amine **a-f** (1.2 eq) and DIEA (1.5 eq) were added. After 30 min the mixture was concentrated under reduced pressure, taken-up with AcOEt (40 mL/mmol boronic acid), washed with 2.5% KHSO_4_ (6×10 mL/mmol boronic acid), H_2_O (2×12 mL/mmol boronic acid) and brine (12 mL/mmol boronic acid), dried over Na_2_SO_4_, filtered and evaporated to dryness under reduced pressure. The crude aryl boronic intermediate **14a-f** was purified by preparative HPLC.

**Step B2.** Aryl bromide **x1-12**, the aryl boronic acid derivative **14a-f** obtained in the previous step (1.05 eq), Na_2_CO_3_ (2 eq) and *N*-tetrabutylammonium bromide (TBAB, 0.01 eq) were given in this order in a screw-cap reactor. H_2_O (2.0 mL/mmol **x1-12**) and 10 mM Pd(EDTA) solution (0.3 mL/mmol aryl **x1-12**) were added, the reactor was closed tightly and heated to 100°C under stirring. After 5 h the mixture was cooled to room temperature, diluted with AcOEt (40 mL/mmol **x1-12**), washed with 5% NaHCO_3_ (15 mL/mmol **x1-12**), H_2_O (15 mL/mmol **x1-12**), 5% KHSO_4_ (15 mL/mmol **x1-12**) and brine (15 mL/mmol **x1-12**), dried over Na_2_SO_4_, filtered and evaporated to dryness under reduced pressure. The crude product **(1–12)(a-f)** was purified by preparative HPLC.

#### General strategy C

**Step C1.** To 3-bromobenzoic acid, aryl boronic acid **y1-12** (1.1 eq), K_2_CO_3_ (3 eq) and palladium(0) tetrakis(triphenylphosphine) were given in a screw-cap reactor, a 8:8:1 toluene/EtOH/H_2_O mixture (8.5 mL/mmol 3-bromobenzoic acid) was added, the reactor was closed tightly and heated to 100°C under stirring. After 4 h the mixture was cooled to room temperature, diluted with AcOEt (10 mL/mmol 3-bromobenzoic acid) and extracted with 5% NaHCO_3_ (4×10 mL/mmol 3-bromobenzoic acid). The pooled basic extracts were acidified to pH 3 by dropwise addition of concentrated HCl under stirring, and then extracted with AcOEt (3×10 mL/mmol 3-bromobenzoic acid). The pooled organic extracts were washed with water (10 mL/mmol 3-bromobenzoic acid) and brine (10 mL/mmol 3-bromobenzoic acid), dried over Na_2_SO_4_, filtered and evaporated to dryness under reduced pressure to yield benzoic acid intermediate **m1-12**.

**Step C2.** In a round-bottomed flask HOBt (1.11 eq) and EDC×HCl (1.10 eq) were added to a suspension of the benzoic acid derivative **m1-12** obtained in step *C1* in anhydrous dichloromethane (5 mL/mmol). After complete dissolution secondary amine **a-f** (1.2 eq) and DIEA (1.5 eq) were added. After 30 min the mixture was concentrated under reduced pressure, taken-up with AcOEt (40 mL/mmol **m1-12**), washed with 5% KHSO_4_ (2×15 mL/mmol **m1-12**), H_2_O (12 mL/mmol **m1-12**), 5% NaHCO_3_ (3×12 mL/mmol **m1-12**) and brine (12 mL/mmol benzoic acid), dried over Na_2_SO_4_, filtered and evaporated to dryness under reduced pressure.

#### General strategy D

**Step D1.** Aryl bromide **x1-12**, 3-boronobenzoic acid (1.05 eq), Na_2_CO_3_ (2 eq), TBAB (0.01 eq) were given in a screw-cap reactor. H_2_O (2 mL/mmol **x1-12**) and 10 mM Pd(EDTA) solution (0.3 mL/mmol **x1-12**) were added, the reactor was closed tightly and heated to 100°C under stirring. After 5 hours the mixture was cooled to room temperature, diluted with AcOEt (10 mL/mmol **x1-12**) and extracted with 5% NaHCO_3_ (4×10 mL/mmol **x1-12**). The pooled basic extracts were acidified to pH 3 under stirring by dropwise addition of concentrated HCl and extracted with AcOEt (3×10 mL/mmol **x1-12**). The pooled organic extracts were washed with H_2_O and brine (10 mL/mmol **x1-12** each), dried over Na_2_SO_4_, filtered and evaporated to dryness under reduced pressure to yield benzoic acid intermediate **m1-12**. If necessary, the crude product was purified by preparative-HPLC.

**Step D2.** In a round-bottomed flask, HOBt (1.11 eq) and EDC×HCl (1.10 eq) were added to a suspension of the benzoic acid derivative **m1-12** obtained in step *C1* in anhydrous dichloromethane (5 mL/mmol). After complete dissolution secondary amine **a-f** (1.2 eq) and DIEA (1.5 eq) were added. After 30 min the mixture was concentrated under reduced pressure, taken-up with AcOEt (40 mL/mmol **m1-12**), washed with 5% KHSO_4_ (2×15 mL/mmol **m1-12**), H_2_O (12 mL/mmol **m1-12**), 5% NaHCO_3_ (3×12 mL/mmol **m1-12**) and brine (12 mL/mmol benzoic acid), dried over Na_2_SO_4_, filtered and evaporated to dryness under reduced pressure.

### Materials and permission for biological experiments

Test and reference compounds, if not otherwise stated, were synthesized as described above or obtained of the highest grade available from Sigma-Aldrich (Buchs, Switzerland). Stock solutions (10 mM, 1 mM) were prepared in dimethyl sulfoxide (DMSO). [1,2,6,7-^3^H]-cortisol, [2,4,6,7-^3^H]-estrone and [2,4,6,7-^3^H]-estradiol were obtained from PerkinElmer (Boston, MA, USA) and [1,2-^3^H]-cortisone from American Radiolabeled Chemicals (St.Louis, MO). Cell culture media were purchased from Sigma Aldrich.

The biological experiments were performed under approval by the Bundesamt für Umwelt (BAFU), Switzerland, permission numbers A130932 and A070126.

### Preparation of cell lysates

Human embryonic kidney (HEK-293) cells either stably expressing human 11β-HSD1 and H6PDH (HHH7 clone, [38]) or 11β-HSD2 (AT8 clone, [39]), or transiently expressing human 17β-HSD1 or 17β-HSD2, were cultivated in Dulbecco’s modified Eagle medium (DMEM) containing 4.5 g/L glucose, 10% fetal bovine serum, 100 U/mL penicillin, 0.1 mg/mL streptomycin, 1 × MEM nonessential amino acids, and 10 mM HEPES buffer, pH 7.4. Cells were washed with phosphate-buffered saline (PBS), centrifuged for 4 min at 150 × g, and the cell pellets were snap frozen on dry ice and stored at −80°C until further use.

### Determination of enzyme activity in cell lysates

Cell lysates were resuspended in TS2 buffer (100 mM NaCl, 1 mM EGTA, 1 mM EDTA, 1 mM MgCl_2_, 250 mM sucrose, 20 mM Tris-HCl, pH 7.4), followed by sonication at 4°C using a Hielscher UP50H ultrasonic processor (0.3 cycle, 20 amplitude %, 20 impulses; Hielscher Ultrasonics GmbH, Teltow Germany). Cell lysates, reaction mixture, and either vehicle (DMSO) or inhibitor were incubated for 10 min at 37°C in a final volume of 22.2 μL. The solvent concentration was 0.1%. 17β-HSD1, 17β-HSD2, and 11β-HSD1 activities were measured in presence of 200 nM of the corresponding substrates, *i*.*e*. estrone, estradiol, or cortisone, as well as 450 μM of either NADPH or NAD^+^. 11β-HSD2 activity was measured in the presence of 50 nM cortisol and 500 μM NAD^+^. An amount of 50 nCi of radiolabeled tracer substrate was included in the experiments. Reactions were stopped by adding an excess of unlabeled cortisone and cortisol (1:1, 2 mM, in methanol) for 11β-HSD measurements and estrone and estradiol (1:1, 2 mM in methanol) for 17β-HSD measurements. The steroids were separated by SIL G-25 TLC plates (Macherey-Nagel, Oensingen, Switzerland), using methanol-chloroform (1:9) for cortisone/cortisol and AcOEt-chloroform (1:4) for estrone/estradiol separation. The bands of the corresponding steroids were excised, followed by scintillation counting and calculation of the amount of substrate conversion. Data were obtained from at least three independent measurements. Glycyrrhetinic acid (CTRL 1) was used as positive control for 11β-HSD1 and 11β-HSD2 inhibition, apigenin (CTRL 2) for 17β-HSD1, and compound 22 from Vuorinen *et al*. [40](CTRL 3) for 17β-HSD2 inhibition.

### 11β-HSD1 activity assay in intact cells

HEK-293 cells co-expressing 11β-HSD1 and H6PDH were seeded in poly-L-lysine coated 96 well-plates (30’000 cells/well) and cultivated in DMEM. At 24 h post-seeding, the cells were washed with charcoal-treated serum-free DMEM (cDMEM) and incubated for 3 h in 100 μL cDMEM. Then, the medium was aspirated and replaced by 30 μL cDMEM. 10 μL of inhibitor dissolved in cDMEM were added, followed by pre-incubation for 25 min at 37°C and measurement of enzyme activity by adding 200 nM cortisone containing 50 nCi of radiolabeled tracer. The reactions were terminated after 30 min of incubation, followed by analysis of substrate conversion as described above. Data were obtained from at least three independent measurements.

Human epidermal keratinocytes obtained from CELLnTEC advanced Cell Systems (Berne, Switzerland) were maintained in CnT-Prime medium (http://cellntec.com/products/cnt-pr/#datasheet) at 37°C. Cells were subcultivated before reaching confluence. After reaching 90% of confluence, the keratinocytes were washed twice with PBS prior to change to cortisol-free CnT-Prime medium (custom-made by CELLnTEC). Keratinocytes were incubated with 1000 nM cortisone in the absence or presence of inhibitors. Compound CAS 1009373-58-3 (CTRL 4, Merck Millipore, Darmstadt, Germany) was used as positive control. After 24 h cell culture supernatants were collected and the amount of cortisol produced was measured on a Mulstiskan Ascent plate reader (MTX Lab Systems, Bradenton, FL) using the Cortisol Parameter Assay Kit (R&D Systems, Minneapolis, MN) following the instructions by the manufacturer. Cell viability was assessed using PrestoBlue^®^ Cell Viability Reagent (Invitrogen, Carlsbad, CA) according to the manufacturer’s instructions.

### Experiments with *ex vivo* skin biopsies

*Ex vivo* skin experiments were performed as contract research by Cutech Biotechnology following the Helsinki declaration and who obtained permission as well as informed consent from the patient (Padova, Italy). Human skin from abdominal plastic surgery of a female donor was used. Skin samples were cut in pieces of approximately 8 × 3 mm (diameter × thickness) and cultured in an air-liquid interface in a perforated ring of stainless steel in contact with culture medium (modified Williams’ E medium, Sigma Aldrich) up to day 6. The culture medium was renewed on day 0 and day 3. The test compounds were applied topically and renewed daily. The application was performed as follows: skin biopsies were gently cleaned with a cotton pad and 4 μL of 10 μM or 100 μM test compound were applied on top of each piece and covered with a 6 mm (diameter) delivery membrane (CoTran 9728 Controlled Caliper Ethylene Vinyl Acetate Membrane, 3M, St. Paul, MN, USA).

Skin biopsies were subjected to UV irradiation using a BIO-SUN system (Vilber Lourmat, Eberhardzell, Germany). The system had the following specifications: Lamp wave length: UVB 312 nm (range 280–320 nm) and UVA 365 nm (range 355–375 nm), range of measure (irradiance): accuracy and linearity ± 0.2%, range of programmed energy: 0.1 to 99.99 J/cm^2^, intensity: UVA (365 nm) 5 mW/cm^2^, UVB (312 nm) 3 mW/cm^2^, UVA + UVB: 5.6 + 3.2 mW/cm^2^, respectively. The selected UV irradiation intensities were 40% and 80% of the Biological Effective Dose (BED) of 7.5 J/cm^2^ UV daylight as described by Del Bino *et al*. [41]. UV 40% BED = 3.0 J/cm^2^ (= 0.1 J/cm^2^ UVB + 2.9 J/cm^2^ UVA); UV 80% = 6.0 J/cm^2^ (= 0.2 J/cm^2^ UVB + 5.8 J/cm^2^ UVA).

Skin viability was assessed in two skin biopsies after 6 days of treatment using methylthiazolyldiphenyl‐tetrazolium bromide (MTT, Sigma Aldrich) staining. Total dermal collagen in skin sections was semi-quantitatively evaluated by Picrosirius Red histochemical staining (using Picric Acid, Fast Green FCF and Direct Red from Sigma Aldrich) that dyes collagen fibers in purple-red. The papillary dermis was selected for the analysis, being the portion of the dermis that shows highest collagen variation in response to treatment. The evaluation was performed by estimating both color intensity and distribution using IMAGE J (NIH) analysis software, allowing to obtain a semi-quantitative evaluation of dermal collagen (collagen score). Two slides of each skin sample were processed by image acquisition and related analysis (*i*.*e*. 12 images for each treatment).

For statistical analysis, one-way ANOVA with permutation test was performed, followed by Tukey’s permutation test.

### Molecular modeling

Protein (PDB code: 2IRW) was retrieved from RSCB database [42] and prepared using the protein preparation wizard from Maestro version 10.4, Schrödinger, LLC, New York, NY, 2015. In short, bond orders were set, hydrogens were added, disulfide bridges were created, all waters were removed and hydrogen bonds were optimized using the automated procedure. Compounds were docked using Glide (version 6.9, Schrödinger, LLC, New York, NY, 2015); two hydrogen bond constrains for Tyr^183^ and Ser^170^ were used. The PyMOL Molecular Graphics System, Version 1.8.0.4 Schrödinger, LLC was used to prepare S2 Fig.

## Results and discussion

### Synthesis of the inhibitors

The biaryl amide scaffold was chosen upon exploring the chemical space of the 11β-HSD1 substrate binding pocket by molecular modeling. All compounds of the designed library consist of a constant benzoyl moiety bound to a cyclic secondary amine via an amide bond and substituted in position 3 with an aromatic moiety (benzene or pyridine derivative) (Fig 1). All derivatives were prepared by the same two reaction steps, namely Suzuki-Miyaura cross-coupling between the constant benzoyl moiety and one of the variable aromatic moieties, and amide formation between the constant benzoyl moiety and a cyclic secondary amine. In principle, there is no obvious advantage in performing either step first; moreover, in the Suzuki-Miyaura cross-coupling step, it is indifferent which aromatic partner is employed as an aryl bromide and which as an aryl boronic acid: as a result, four alternative synthesis strategies are possible for each library compound (Fig 2).

In the view of future developments, the practicability of the four strategies was compared. Each of the four routes was applied to prepare at least one of the designed compounds (Fig 2). All four synthesis routes gave satisfactory results in terms of yields, but strategy B (based on amide formation between 3-boronobenzoic acid and secondary amine **a-f** followed by Suzuki-Miyaura cross-coupling with aryl bromide **x1-12**) was less practical than the remaining approaches, due to the need for chromatographic purification after amide formation, whereas the alternative routes generally allow to obtain the product in pure form after simple extractive work-up. Also, different literature protocols were tested for the Suzuki-Miyaura cross-coupling step [37, 43, 44], whereas a standard solution-phase peptide chemistry method employing 1-hydroxybenzotriazole (HOBt) and water-soluble carbodiimide (EDC×HCl) was employed for all amide formation steps. Regarding the reaction protocols for Suzuki-Miyaura cross-coupling, that of Korolev et al. [37] yielded particularly good results in terms of low loading of air-stable catalyst and very low amounts by-products formed. A schematic overview of the synthesized compounds **1b** to **12e** is given in S1 Fig.

### Analysis of biaryl amide derivatives inhibiting 11β-HSD1 measured in cell lysates

A total of 22 compounds containing a biaryl amide core and differing in their variable ring substituents (R1) and the cyclic secondary amine substituent (R2) were screened for inhibition of 11β-HSD1 and 11β-HSD2 activity measured in cell lysates in the presence of 100 nM or 1 μM of the respective compound, using glycyrrhetinic acid (GA, CTRL 1) as a positive control. For the most active compounds IC_50_ was determined using eight different inhibitor concentrations (an example is shown in S2 Fig).

**Fig 3:**
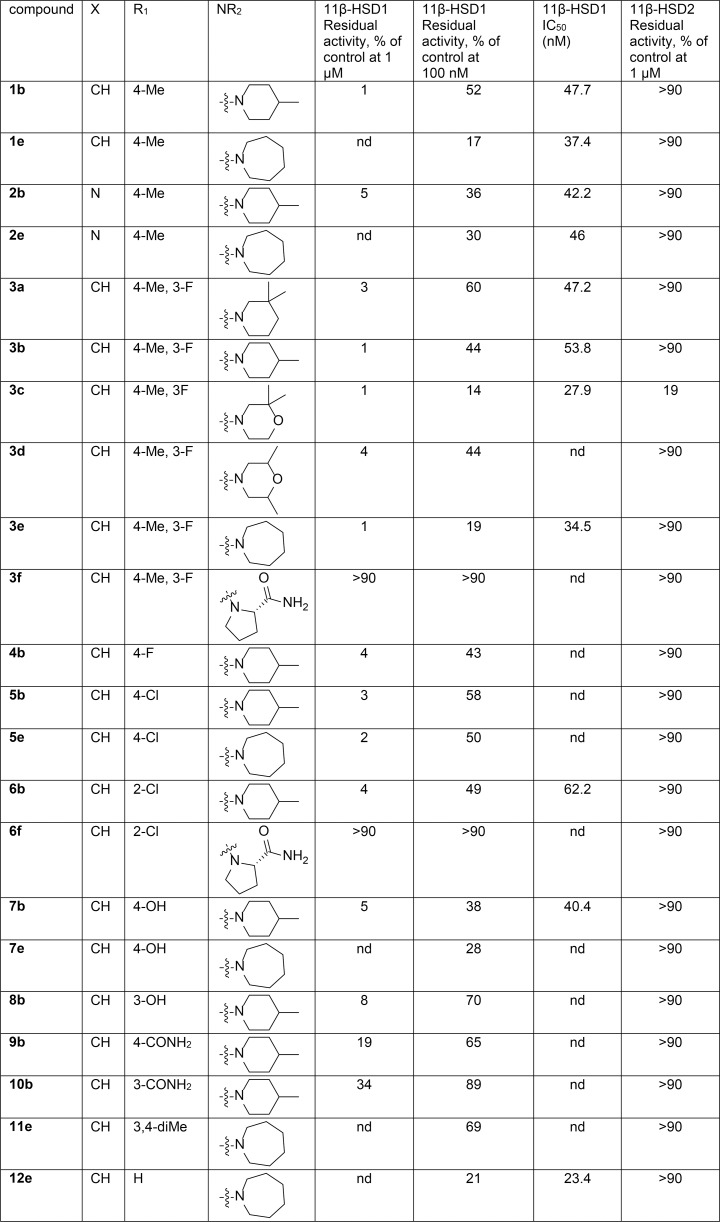
Activity of synthesized compounds toward 11β-HSD1 and 11β-HSD2. Enzyme activity was measured in lysates of HEK-293 cells expressing recombinant human 11β-HSD1 or 11β-HSD2 as described in Materials and Methods. Data represent mean from three independent experiments. nd: not determined.

In a first step, the activities of six compounds bearing a 4-methyl, 3-fluoride substituent R1 and with different cyclic secondary amine R2 substituents were compared. Compounds **3a-e** were very potent 11β-HSD1 inhibitors showing more than 95% inhibition at a concentration of 1 μM and at least 40% inhibition at 100 nM (Fig 3). Interestingly, the introduction of an azepane ring slightly increased activity (compare **3a** with **3e**) while retaining selectivity, which prompted us to expand the structure activity relationship (SAR) data around the azepane derivatives at a later step.

In this first series (**3a-f**) only the introduction of a prolinamide group (**3f**) abolished the activity towards 11β-HSD1. Visualization of the docked pose of prolinamide compound **3f** revealed that in order to form the expected hydrogen bonds with residues Ser^170^ and Tyr^183^ a negative steric interaction with Ile^121^ in the binding site arises, and there are no stabilizing interactions from the prolinamide group (no apparent H-bonds), which may explain the lack of activity of these compounds (for illustration see S3 Fig). Also, the prolinamide derivative **6f** with a chlorine in 2-position as R1 substituent was inactive towards 11β-HSD1 at 1 μM, for similar reasons as for **3f**.

In a second step, the influence of R1 variations was analyzed while keeping constant either the 4-methyl piperidine or the azepane moiety. In most cases the exchange of the substituent in the variable aryl group did not induce a large difference in activity (no greater than a factor of two). However, the introduction of a carboxamide group in **9b** and **10b** either at the 3- or the 4-position resulted in substantial loss of activity. Also the introduction of a 3-hydroxy (**8b**) or a 3,4-dimethyl (**11e**) substituent resulted in lower activity.

Among all compounds tested, **3c** was the only one to show slight inhibition of 11β-HSD2 with 81% residual activity at 1 μM. Among the most potent and selective compounds two 4-methylpiperidine derivatives (**2b**, **7b**) and two azepane derivatives (**3e**, **12e**) were chosen for further investigation.

### Selectivity of 11β-HSD1 inhibitors over 17β-HSD1 and 17β-HSD2

Since 17β-HSD1 and 17β-HSD2 have important roles in the modulation of active estrogens and androgens and they share structural similarity with 11β-HSD1 and 11β-HSD2, respectively [45], the four selected compounds were tested for inhibitory activity against these enzymes. None of the four compounds inhibited 17β-HSD1 or 17β-HSD2 at a concentration of 1 μM, in contrast to the positive controls apigenin (CTRL 2) and compound 22 of Vuorinen et al. [40] (CTRL 3), respectively (S4 Fig).

The short-chain dehydrogenase/reductase (SDR) family comprises more than 80 members in the human genome [46]. To further assess the selectivity of the newly identified compounds, additional SDRs that are expressed in skin should be included in the testing in order to avoid, among others, the inhibition of 3β-HSD involved in steroidogenesis [7, 47], or retinol dehydrogenases involved in retinoid metabolism or in the detoxification of unsaturated aldehydes formed upon oxidative stress [48].

### Inhibition of 11β-HSD1 in intact cells

**Fig 4:**
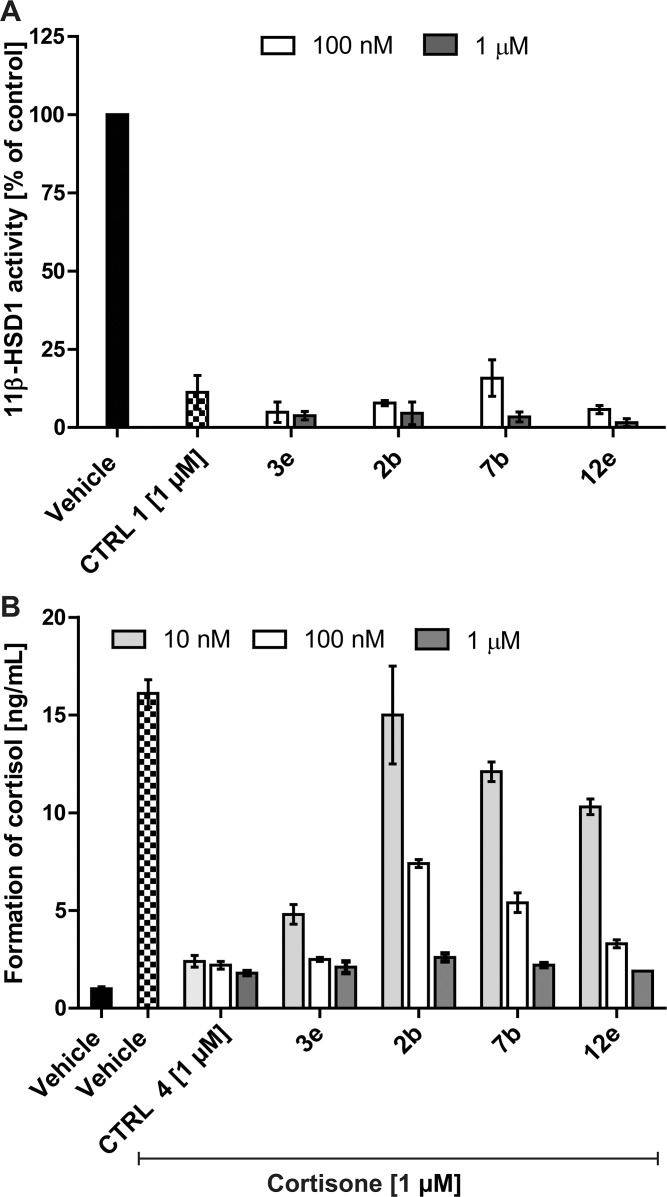
Inhibition of 11β-HSD1 activity in intact cells. (A), Inhibition of 11β-HSD1-dependent cortisol formation in intact HEK-293 cells. HEK-293 cells stably co-expressing human 11β-HSD1 and H6PDH were incubated for 30 min in the presence of 200 nM radiolabeled cortisone and either 1 μM of the positive control inhibitor glycyrrhetinic acid (CTRL 1) or 100 nM and 1 μM of the respective test compounds. Formation of cortisol was determined by separation of steroids by TLC and scintillation counting. Data represent mean ± SD of three independent experiments, each performed in triplicate. (B), Inhibition of 11β-HSD1-dependent cortisol formation in intact human keratinocytes. Primary human keratinocytes were grown for two days, followed by incubation for 24 h with 1 μM of cortisone and various concentrations of inhibitor. Compound CAS 1009373-58-3 (Merck, CTRL 4) was used as positive control. An additional vehicle control was measured in the absence of exogenous cortisone (black bar). Formation of cortisol was determined by an enzyme immune assay. Data represent mean ± SD from three experiments. Shapiro-Wilk test was used to assess the normality of data. One-way analysis of variance (ANOVA) and Dunnett's multiple-comparison test were performed to evaluate differences between inhibitor treatments compared to the solvent control. All values were significantly different (p<0.01) compared to vehicle control in the presence of cortisone, except treatment of keratinocytes with 10 nM **2b**.

The four inhibitors **2b**, **3e**, **7b** and **12e** were tested for their activity in intact HHH7 cells stably expressing 11β-HSD1 and H6PDH [38]. Cells were incubated at a final concentration of 200 nM cortisone in the presence of 1 μM GA (CTRL 1) as positive control or 100 nM and 1 μM of the test compounds (Fig 4A). All compounds almost completely inhibited 11β-HSD1 at both concentrations tested, demonstrating that effective concentrations were reached at the active site of the enzyme in the endoplasmic reticulum.

Next, the activity of the four selected compounds as inhibitors of the 11β-HSD1-mediated cortisol production in primary human keratinocytes was determined. Keratinocytes were incubated for 24 h with 1 μM cortisone and the respective inhibitor. Compound CAS 1009373-58-3 (CTRL 4, at 1 μM) was used as positive control. At a concentration of 1 μM all compounds almost completely inhibited cortisol formation, and the estimated IC_50_ values were 100 nM or lower (Fig 4B). The potency of inhibition was **3e** > **12e** > **7b** > **2b**. Thus, all four compounds efficiently blocked cortisol formation in primary human keratinocytes.

### *Ex vivo* human skin experiments

To prepare for topical applications, experiments with skin biopsies were performed. First, UV irradiation strength to induce moderate skin damage and potential toxic effects of the selected inhibitors was assessed. Exposure of the skin samples to UV at 3.0 J/cm^2^ and 6.0 J/cm^2^ resulted in a progressive decrease in viability to 90% and 72% (significant compared with unexposed control, p<0.001), respectively (S5 Fig). Importantly, the addition of inhibitors did not affect skin viability, thus allowing studying effects of these inhibitors on collagen content in UV-exposed skin.

**Fig 5:**
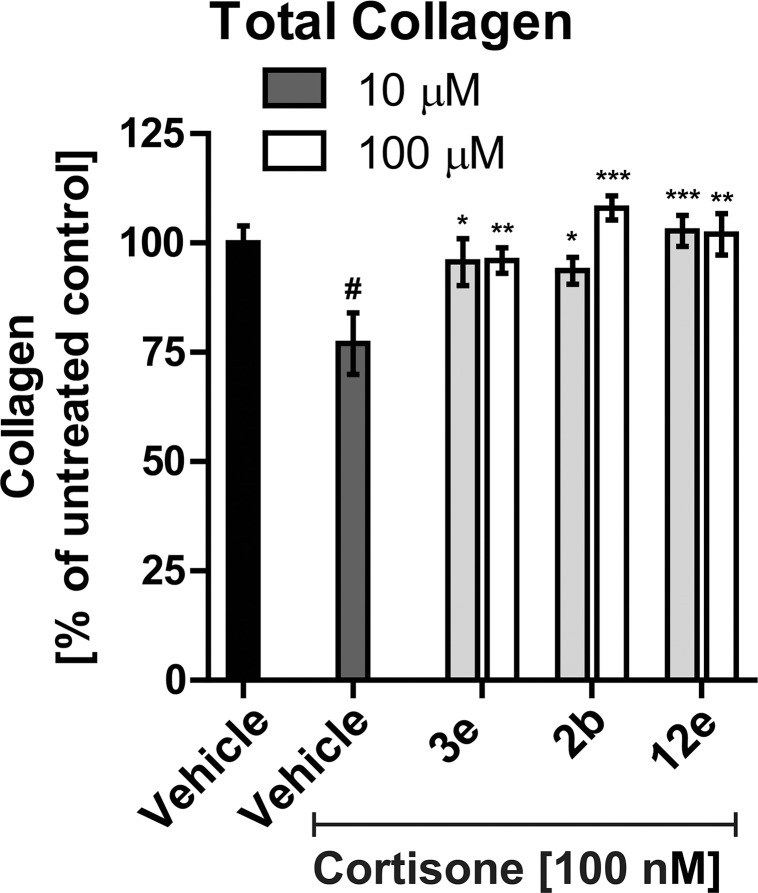
Reversal of cortisone-mediated decrease in dermal total collagen content upon treatment with selected compounds. Human skin biopsies were treated topically with 4 μL of 10 μM or 100 μM of the selected compounds for 6 days. After the first 24 h of incubation with the inhibitors, the skin samples were simultaneously treated with inhibitor and 100 nM cortisone for the remaining 5 days. Dermal collagen content was semi-quantitatively assessed by Picrosirius Red histochemical staining. Data represent mean ± SEM from 6 human biopsies. Shapiro-Wilk test indicated that the data followed a normal distribution and unpaired t-test was used to test for significant differences. # *p*<0.01 compared to vehicle control in the absence of cortisone; * *p*<0.05, ** *p*<0.01, *** *p*<0.001 compared to cortisone treated vehicle control.

Next, the effect of cortisone, which requires 11β-HSD1-dependent conversion to cortisol, on skin viability and collagen content was analyzed. Treatment of skin biopsies with cortisone did not significantly lower skin viability despite a trend decrease by 10–15% at 100 nM (S6A Fig). In contrast, dermal collagen was significantly decreased upon exposure to 10 nM, 100 nM and 1 μM of cortisone by 20–26% (p<0.01, S6B Fig). The simultaneous treatment with 100 nM cortisone and either 10 μM or 100 μM of inhibitor (**3e**, **2b**, or **12e**) prevented the cortisone-induced collagen decrease (Fig 5).

**Fig 6:**
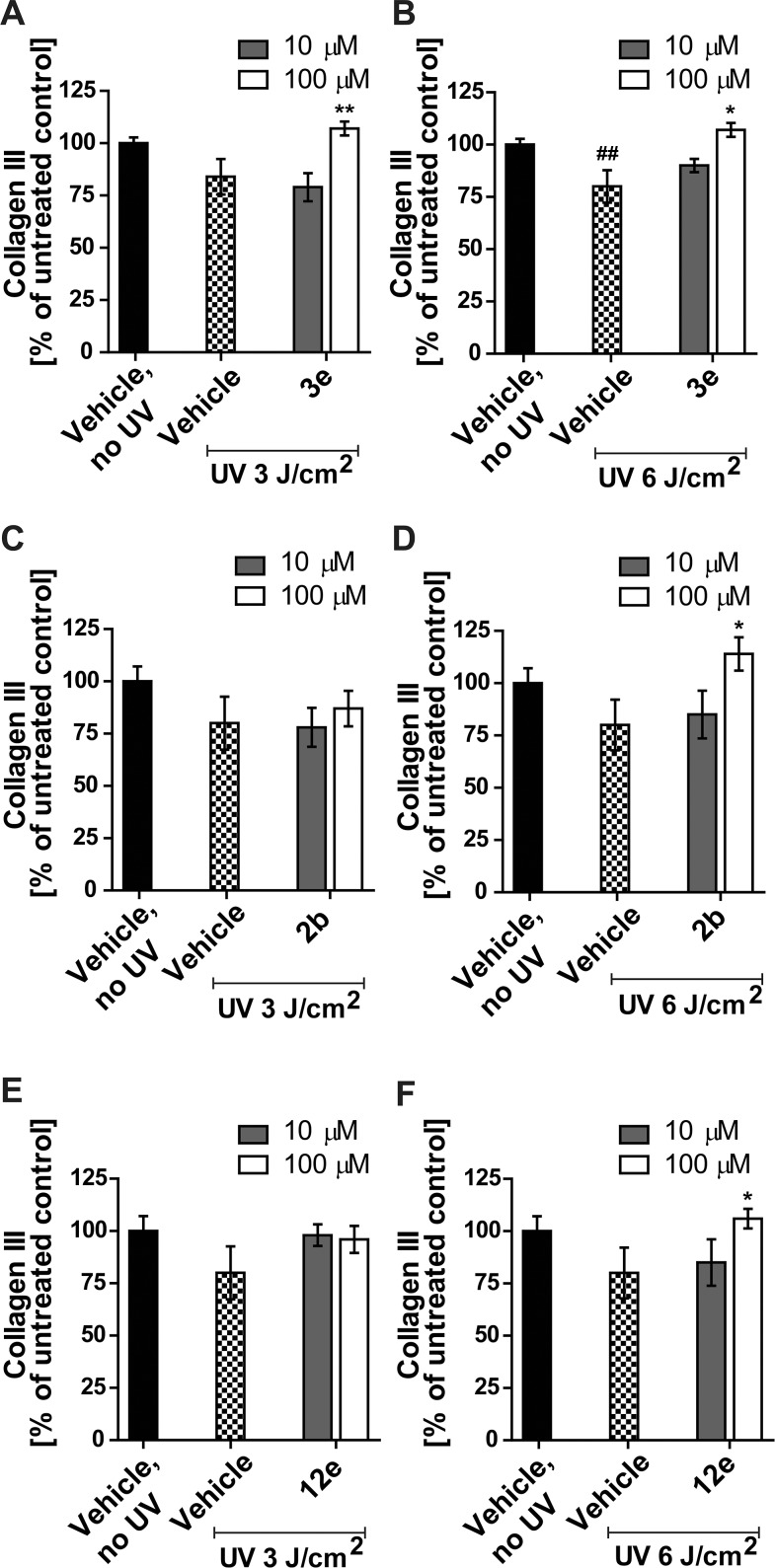
Effect of selected compounds on dermal collagen III content in UV exposed human skin samples. Human skin biopsies were treated topically with 4 μL of 10 μM or 100 μM of compound **3e** (A,B), **2b** (C,D), or **12e** (E,F) and exposed to 3.0 J/cm^2^ (A,C,E) or 6.0 J/cm^2^ UV irradiation (B,D,F) for 6 days. Dermal collagen III expression was detected using a mouse monoclonal anti-collagen III antibody. Data represent mean ± SEM. Per treatment 12 skin samples were analyzed. The data set presented a normal distribution using Shapiro-Wilk test and an unpaired t-test was used to test for significant differences. ## *p*<0.01 for UV exposed vehicle control *vs* non-irradiated vehicle control. * *p*<0.05, ** *p*<0.01 for UV exposed inhibitor treated *vs* UV exposed vehicle control.

Furthermore, in a preliminary set of experiments, skin biopsies were UV irradiated at 3.0 J/cm^2^ and 6.0 J/cm^2^ and treated topically with 4 μL of 10 μM or 100 μM of the respective inhibitor, followed by determination of collagen III content using a mouse monoclonal anti-collagen III antibody (Sigma Aldrich). A comparison of the UV exposed vehicle control with the non-irradiated vehicle control revealed a significant decrease in collagen III by about 20% (p<0.01) in Fig 6B but only a trend in Fig 6A and 6C–6F. Treatment with compound **3e** prevented the decrease in collagen III levels, with significantly higher collagen III at 100 μM upon exposure to 3.0 J/cm^2^ UV irradiation (p<0.01) and 6.0 J/cm^2^ UV irradiation (p<0.05). In the group of biopsies exposed to 6.0 J/cm^2^ UV irradiation treatment with 100 μM of compounds **2b** or **12e** resulted in significantly higher collagen III levels compared with the UV treated vehicle control. In contrast, treatment with 10 μM of **2b** or **12e** did not result in significant changes. These initial experiments shown in Figs 5 and 6 suggest that inhibition of 11β-HSD1 can prevent the decrease of collagen upon exposure of skin biopsies to cortisone and/or UV irradiation.

Nevertheless, future experiments should include time-dependence of UV exposure and 11β-HSD1 inhibitor treatment as well as investigations into gene expression to assess the potential of such compounds in the prevention of skin damage and skin aging. An increased expression of 11β-HSD1 upon exposure of skin to UV irradiation was observed previously [14]. Thus, inhibition of 11β-HSD1 might be beneficial in situations of chronically elevated glucocorticoid activation in skin to avoid related adverse effects. Besides, CYP11B1 inhibitors might represent an alternative approach to avoid excessive local glucocorticoid effects due to enhanced steroidogenic cortisol production upon exposure to UV irradiation [16, 17]. It will also be important to assess the expression levels and sensitivity to cortisol of GR and MR in skin. Exposure to UV irradiation lowered epidermal GR expression [14]. Whether this results in a shift from GR to MR response by glucocorticoids as observed in microglial cells [49] remains to be investigated.

## Conclusion

The present study describes novel biaryl amide compounds prepared by Suzuki-Miyaura cross-coupling. The derivatives were first screened for inhibition of 11β-HSD1 and 11β-HSD2 activity measured in cell lysates at concentrations of 1 μM and 100 nM, followed by determination of IC_50_ for the most active compounds. Interestingly, the introduction of an azepane ring slightly increased the inhibitory activity (compare **3a** with **3e**) while retaining selectivity, which prompted us to expand the SAR data around the azepane derivatives in a next step. Among the most potent and selective compounds two 4-methylpiperidine derivatives (**2b**, **7b**) and two azepane derivatives (**3e**, **12e**) were chosen for further investigation as they were also found to be selective against 17β-HSD1 and 17β-HSD2 at a concentration of 1 μM. The four selected inhibitors **2b**, **3e**, **7b** and **12e** were tested for their activity in intact HHH7 cells stably expressing 11β-HSD1 and H6PDH [38]. All four compounds selectively inhibited 11β-HSD1 in the nanomolar range and efficiently blocked cortisol formation in intact primary human keratinocytes. Three compounds, **2b, 3e,** and **12e**, were further shown to prevent the cortisone-dependent decrease in collagen content in human skin biopsies. Furthermore, the results from UV irradiated skin biopsies suggest that these compounds may also reduce the decrease in collagen III content observed in UV exposed skin.

Clearly, further experiments are needed to assess time- and dose-dependent effects of the selected inhibitors and to optimize UV exposure as well as test the inhibitors at combined treatment with cortisone and UV exposure. Furthermore, the impact of topical administration of 11β-HSD1 inhibitors on dermal thickness, number of dermal fibroblasts and keratinocytes in human skin has to be evaluated, in analogy to the observations in mice [31, 33]. Since 11β-HSD1 expression in skin has been shown to increase with age and excessive cortisol levels result in skin atrophy [13, 32], topical application of 11β-HSD1 inhibitors may represent a promising approach to prevent skin damage and delay skin aging.

## Supporting information

S1 Fig Overview of the synthesized compounds. (TIF)

S2 Fig IC_50_ for 11β-HSD1 of the selected compounds 2b, 3e, 7b and 12e. The selected test compounds at different concentrations were analyzed for their ability to inhibit 11β-HSD1-dependent conversion of 200 nM cortisone to cortisol. IC_50_ was calculated for compound **2b** (A), **3e** (B), **7b** (C) and **12e** (D) from results obtained from three independent experiments.
(TIF)

S3 Fig Binding site surfaced view of the docked pose of prolinamide 3f (orange). For clarity only residues that have major interactions with the ligand are shown. The negative steric interaction with Ile^121^ is indicated by a red dotted line. The interactions with Ser^170^ and Tyr^183^ are shown by yellow dotted lines.
(TIF)

S4 Fig Selectivity of the 11β-HSD1 inhibiting test compounds over 17β-HSD1 and 17β-HSD2. The selected test compounds at a concentration of 1 μM were analyzed for their ability to inhibit 17β-HSD1-dependent conversion of 200 nM estrone to estradiol (A) and the 17β-HSD2-dependent conversion of 200 nM estradiol to estrone (B). Apigenin (CTRL 2) and compound 22 of Vuorinen et al. [22] (CTRL 3) served as positive controls. Data represent mean ± SD from three independent experiments.
(TIF)

S5 Fig Impact of UV irradiation and selected compounds on cell viability in human skin biopsies. The experiments with human full skin biopsies were performed by Cutech Biotechnology. Skin samples were treated topically with vehicle or the respective compounds (4 μL of 10 μM or 100 μM compound applied on top of each biopsy specimen) for 6 days either in the absence of UV treatment or upon exposure to 3.0 J/cm^2^ or 6.0 J/cm^2^ UV irradiation. Cell viability was determined after 6 days using MTT. Data represent mean ± SEM from 6 human biopsies.
(TIF)

S6 Fig Effect of cortisone on cell viability in human skin biopsies. Human skin biopsies were treated with 0.01 μM, 0.1 μM or 1 μM cortisone for 6 days, followed by assessment of skin viability using the MTT assay. Data represent mean ± SEM from 6 samples derived from 2 different skin biopsies. ** p<0.01 vs vehicle control.
(TIF)

S1 File Experimental section. Materials and methods for chemistry.
(DOC)
