# Supplementary figures and images for: Novel 11β-hydroxysteroid dehydrogenase 1 inhibitors reduce cortisol levels in keratinocytes and improve dermal collagen content in human ex vivo skin after exposure to cortisone and UV
Source: PLoS One. 2017 Feb 2;12(2):e0171079. doi: 10.1371/journal.pone.0171079 (PMC5289826; doi:10.1371/journal.pone.0171079)

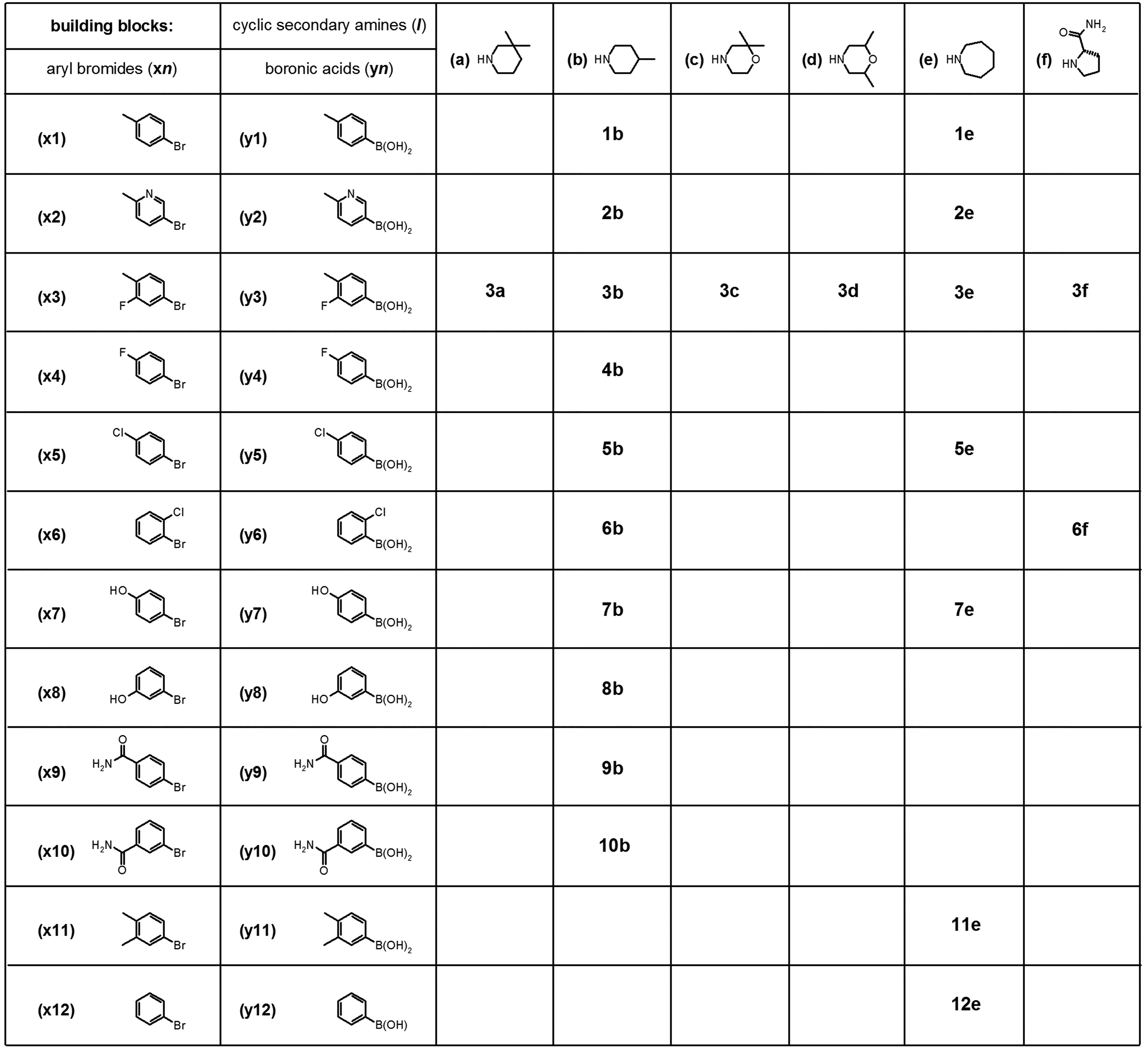

Supplement: S1 Fig — (TIF) [file pone.0171079.s001.tif]

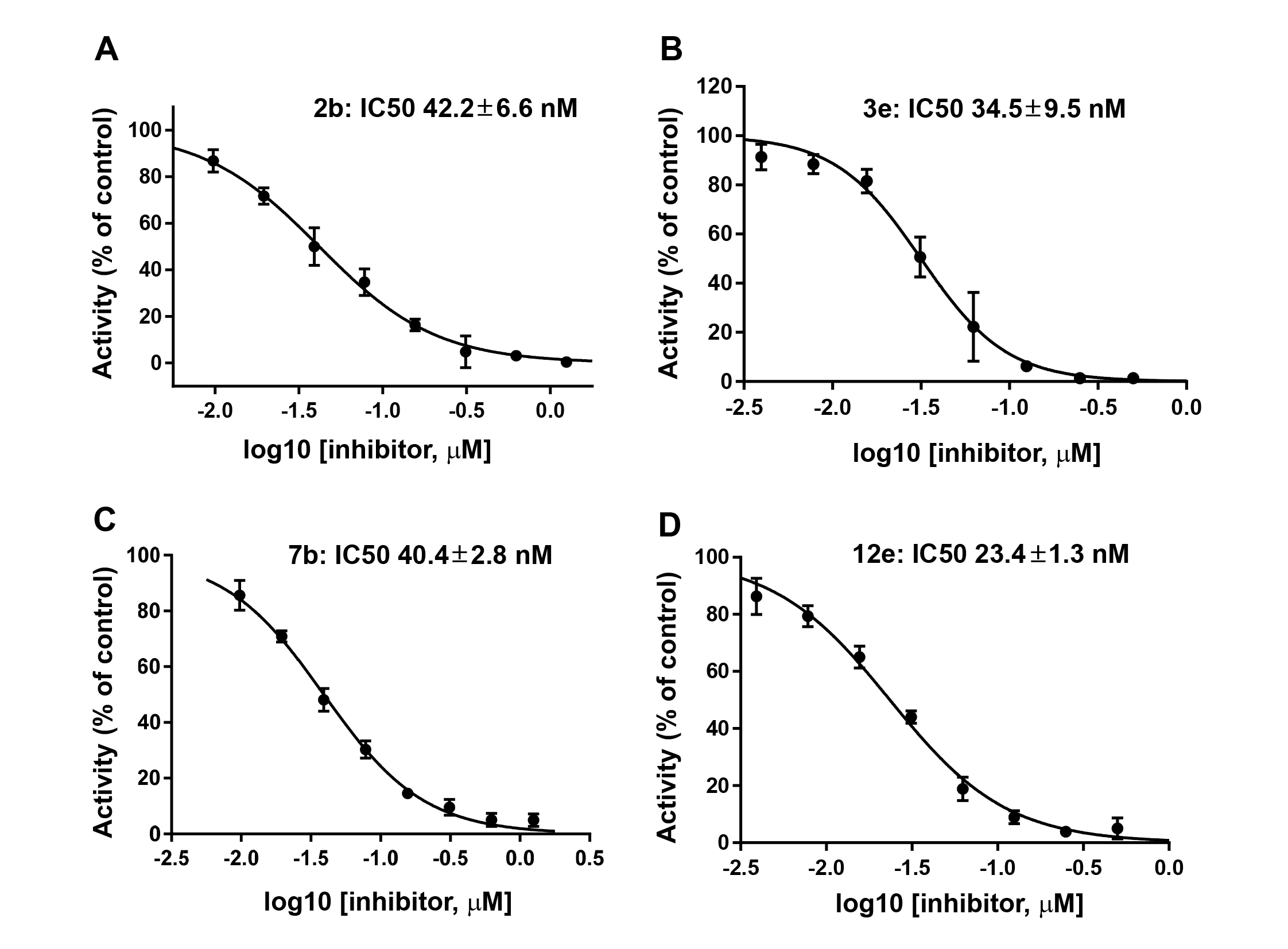

Supplement: S2 Fig — The selected test compounds at different concentrations were analyzed for their ability to inhibit 11β-HSD1-dependent conversion of 200 nM cortisone to cortisol. IC50 was calculated for compound 2b (A), 3e (B), 7b (C) and 12e (D) from results obtained from three independent experiments. (TIF) [file pone.0171079.s002.tif]

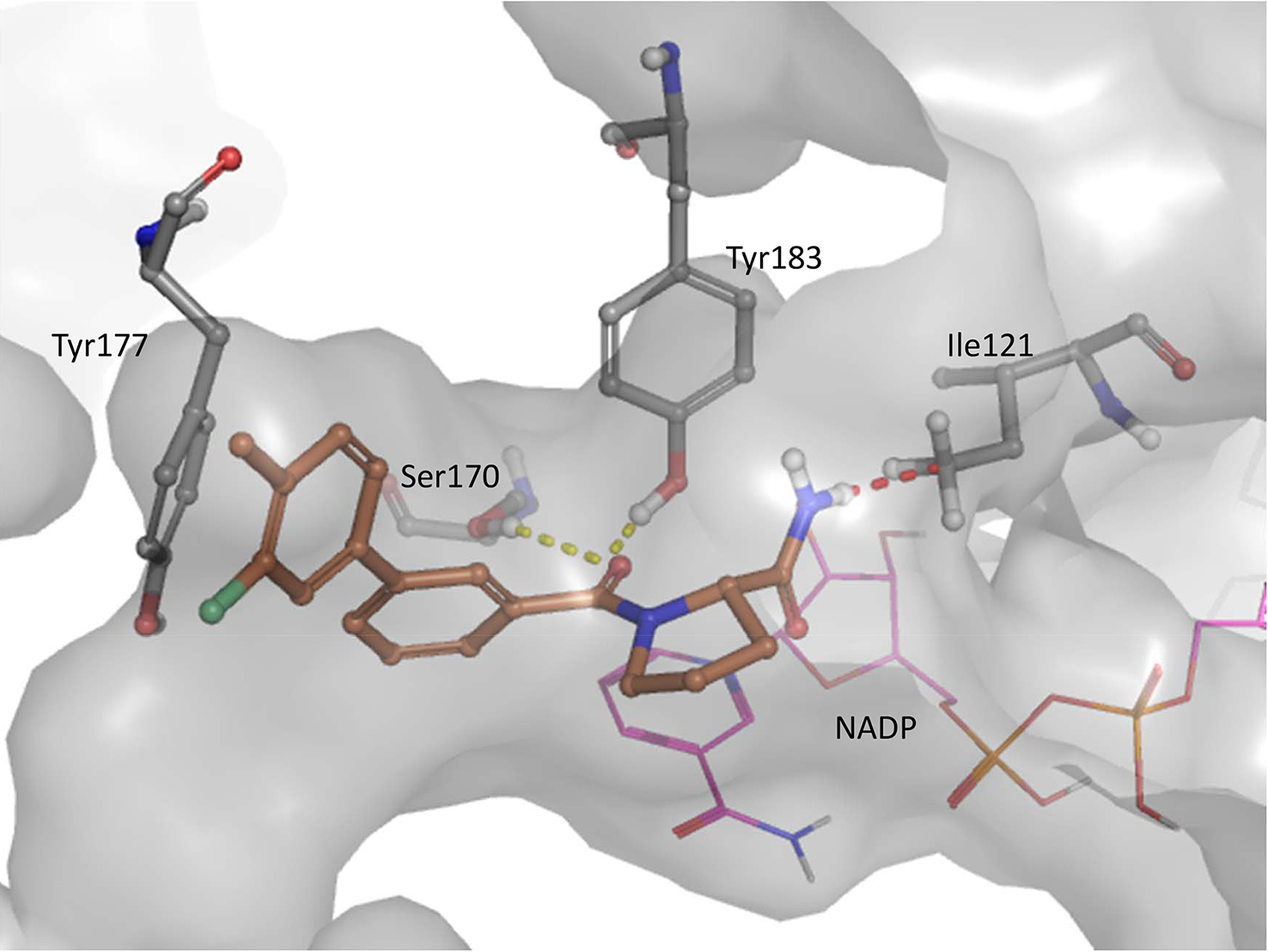

Supplement: S3 Fig — For clarity only residues that have major interactions with the ligand are shown. The negative steric interaction with Ile121 is indicated by a red dotted line. The interactions with Ser170 and Tyr183 are shown by yellow dotted lines. (TIF) [file pone.0171079.s003.tif]

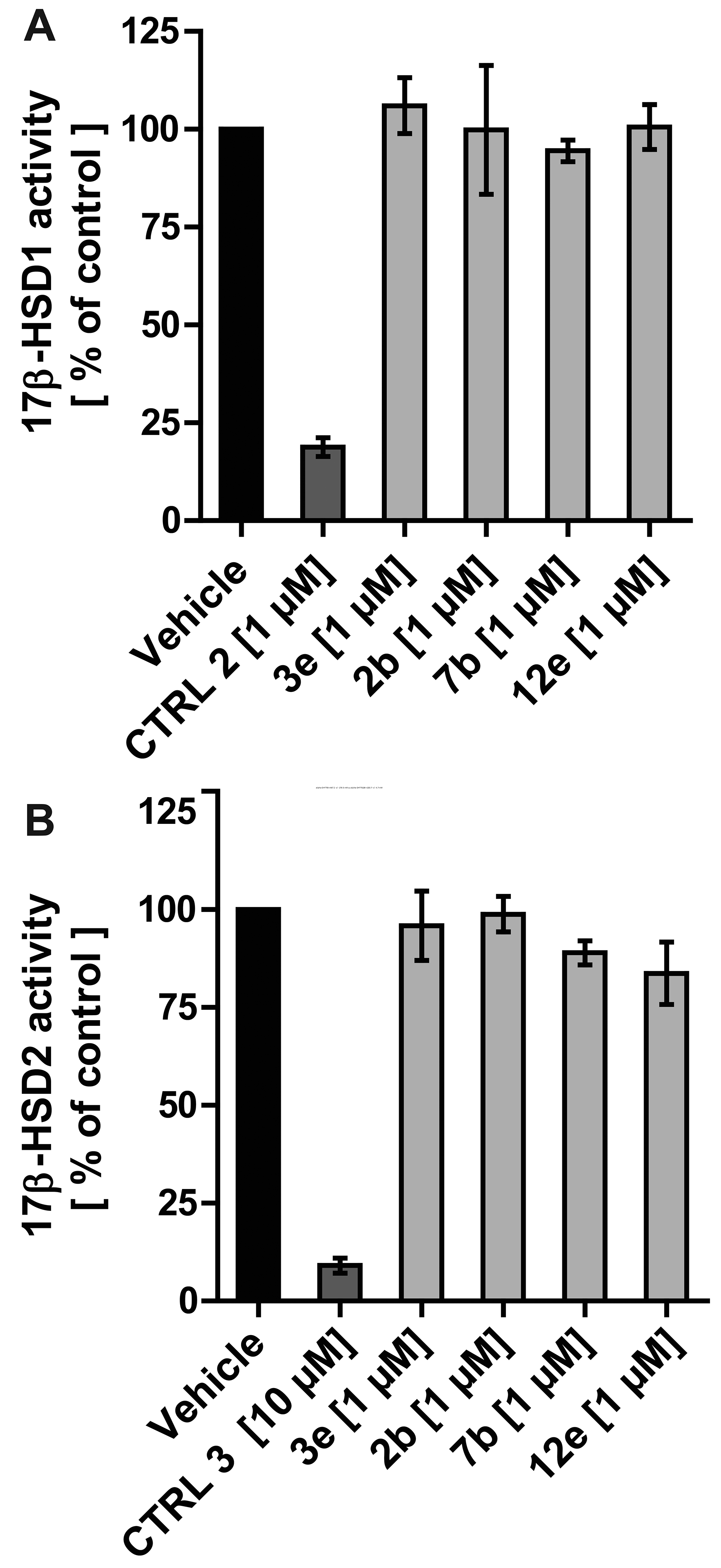

Supplement: S4 Fig — The selected test compounds at a concentration of 1 μM were analyzed for their ability to inhibit 17β-HSD1-dependent conversion of 200 nM estrone to estradiol (A) and the 17β-HSD2-dependent conversion of 200 nM estradiol to estrone (B). Apigenin (CTRL 2) and compound 22 of Vuorinen et al. [22] (CTRL 3) served as positive controls. Data represent mean ± SD from three independent experiments. (TIF) [file pone.0171079.s004.tif]

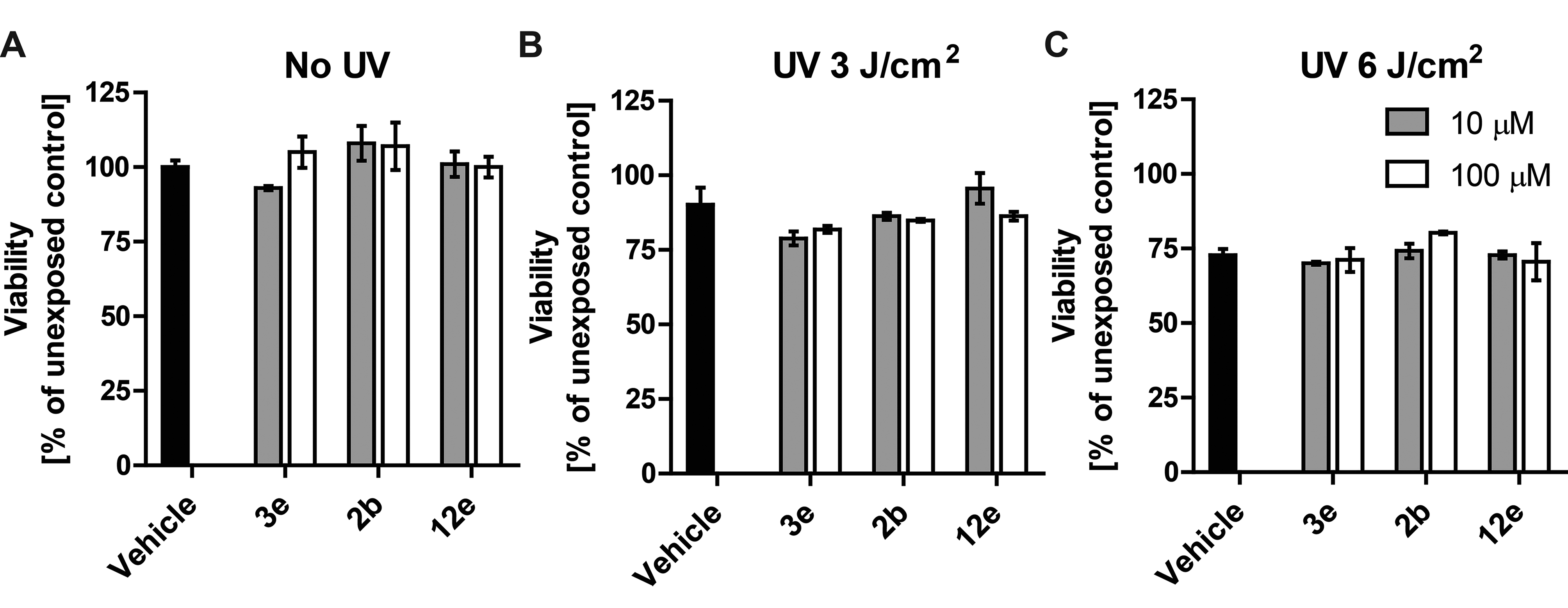

Supplement: S5 Fig — The experiments with human full skin biopsies were performed by Cutech Biotechnology. Skin samples were treated topically with vehicle or the respective compounds (4 μL of 10 μM or 100 μM compound applied on top of each biopsy specimen) for 6 days either in the absence of UV treatment or upon exposure to 3.0 J/cm2 or 6.0 J/cm2 UV irradiation. Cell viability was determined after 6 days using MTT. Data represent mean ± SEM from 6 human biopsies. (TIF) [file pone.0171079.s005.tif]

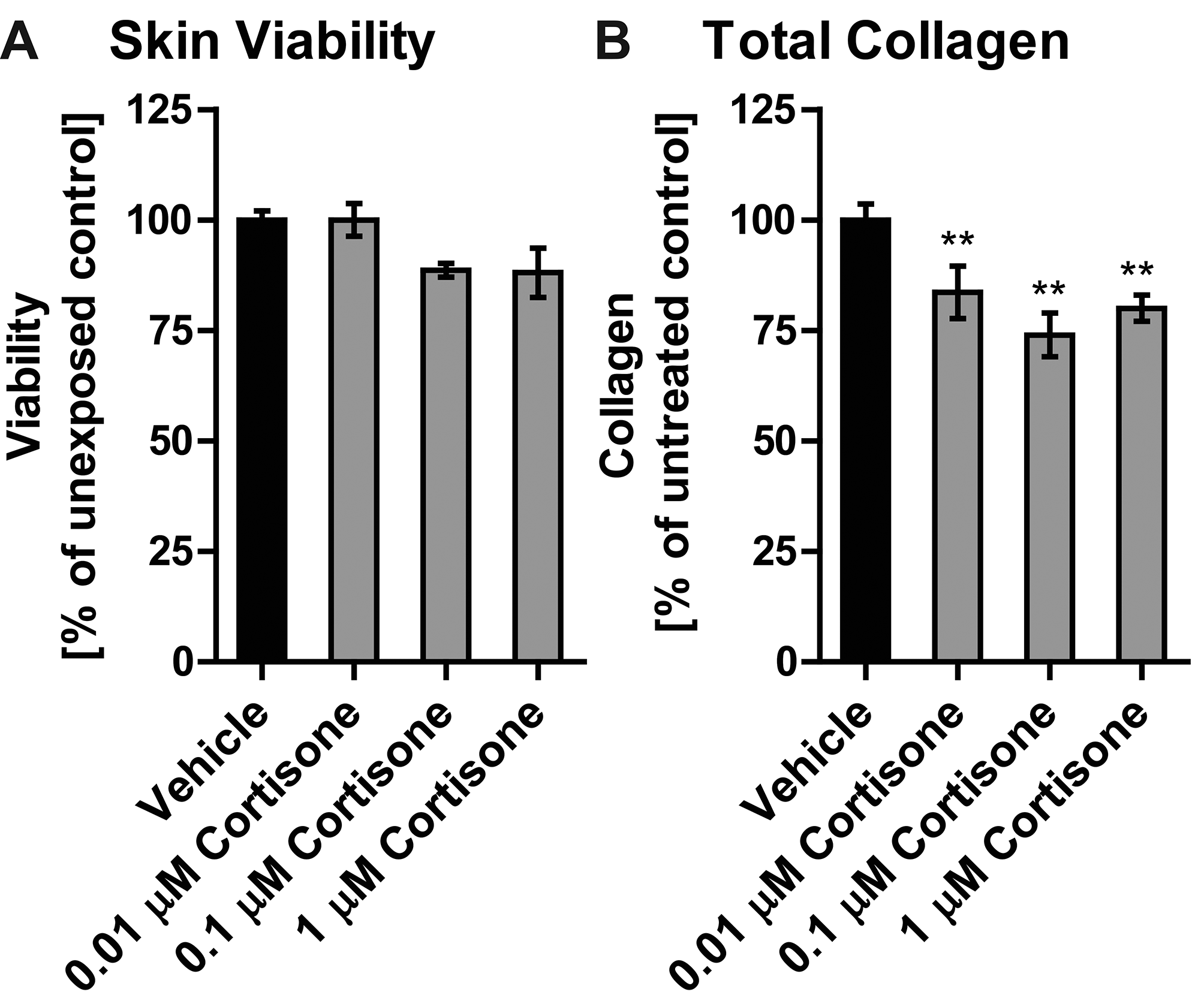

Supplement: S6 Fig — Human skin biopsies were treated with 0.01 μM, 0.1 μM or 1 μM cortisone for 6 days, followed by assessment of skin viability using the MTT assay. Data represent mean ± SEM from 6 samples derived from 2 different skin biopsies. ** p<0.01 vs vehicle control. (TIF) [file pone.0171079.s006.tif]
