# Supplementary material for: Novel 11β-hydroxysteroid dehydrogenase 1 inhibitors reduce cortisol levels in keratinocytes and improve dermal collagen content in human ex vivo skin after exposure to cortisone and UV
Source: PLoS One. 2017 Feb 2;12(2):e0171079. doi: 10.1371/journal.pone.0171079 (PMC5289826; doi:10.1371/journal.pone.0171079)
Supplement: S1 File — Materials and methods for chemistry. (DOC) [file pone.0171079.s007.doc]

S1 File

Experimental section

Material and methods for chemistry

Dichloromethane for amidation reactions was dried over sodium sulfate and stored under argon. Diethyl ether was dried over phosphoric anhydride and stored under argon. All other reagents were reagent or analytical grade and used as received. All air- and water- sensitive reactions, including Suzuki-Miyaura cross-couplings, were performed under argon. milliQ H_2_O for cross-coupling reactions was degassed by sparging with argon under vacuum for 30 min prior to use. The catalytic 10 mM Pd(EDTA) solution was prepared from palladium(II) chloride, ethylenediaminetetraacetic acid (EDTA) disodium salt dihydrate and sodium carbonate as described in the literature [1].

Melting points were measured on a Büchi Melting Point B-540 instrument and are uncorrected. Preparative purifications were performed on a Waters High Performance Liquid Chromatography LC-2525 equipped with a Waters 2767 Sample Manager and a Waters FCII automated fraction collector, using a Grom Saphir 110 C18 10 μm 50×300 mm^2^ preparative column and a Waters 2487 double wavelength UV-Vis detector operating at 220 and 254 nm. H_2_O + 0.07% TFA and acetonitrile + 0.07% TFA were used as eluents, with a flow of 55 mL/min. Nuclear magnetic resonance spectra were recorded on a Bruker Avance 300 spectrometer operating at 300 MHz for ^1^H and 75.5 MHz for ^13^C. Spectra were recorded in deuterochloroform (CDCl_3_) or perdeuterated methyl sulfoxide (*d_6_*‑DMSO) and were referenced to the residual solvent signal. Analytical chromatograms were measured on a Waters Acquity Ultra Performance Liquid Chromatography, equipped with an Acquity HSS T3 100 Å, 1.8 μm 2.1×50 mm^2^ analytical column and a PDA detector operating in the 200-400 nm wavelength range. H_2_O + 0.02% TFA and acetonitrile + 0.02% TFA were used as eluents, with a flow of 0.5 mL/min. Low-resolution mass-spectra were measured on a Waters Acquity I‑Class Ultra Performance Liquid Chromatography, equipped with an Acquity HSS T3 100 Å, 1.8 μm 2.1×50 mm^2^ analytical column and a PDA detector operating in the 200-400 nm wavelength range coupled to a Waters Single Quadrupole Detector mass spectrometer operating in positive electrospray ionization (ESI+) mode and detecting in the *m/z* range 100-1500. H_2_O + 0.04% HCOOH and acetonitrile + 0.04% HCOOH were used as eluents, with a flow of 0.6 mL/min.

Exact mass measurements have been performed by high-resolution mass spectrometry on a Bruker 4G Q-TOF-MS instrument (Bruker Daltonik GmbH, Bremen, Germany). The instrument was equipped with an Apollo II electrospray source operating in positive ionization mode to record [M + H]^+^ pseudomolecular ions.

General synthesis strategies

Where not otherwise stated, the library compounds were prepared by means of one of the following two‑step synthesis strategies.

*General strategy A*

*Step A1*

In a round-bottomed flask 3-bromobenzoic acid was dissolved in anhydrous dichloromethane (5 mL/mmol) under stirring at room temperature and 1‑hydroxybenzotriazole (HOBt, 1.11 eq) and *N*‑(3‑dimethylamino)-propyl-*N’*-ethyl-carbodiimide hydrochloride (EDC×HCl, 1.10 eq) were added. After quantitative activation (as judged by UPLC analysis), the required secondary amine **a-f** (1.2 eq) and *N*-ethyl-di*iso*propylamine (DIEA, 1.5 eq) were added. After 30 min the mixture was concentrated under reduced pressure, taken-up with ethyl acetate (40 mL/mmol 3‑bromobenzoic acid), washed with 5% KHSO_4_ (2×15 mL/mmol 3-bromobenzoic acid), H_2_O (12 mL/mmol 3-bromobenzoic acid), 5% NaHCO_3_ (3×12 mL/mmol 3-bromobenzoic acid) and brine (12 mL/mmol 3-bromobenzoic acid), dried over Na_2_SO_4_, filtered and evaporated to dryness under reduced pressure to yield aryl bromide intermediate **13a-f**.

*Step A2^[1]^*

The aryl bromide derivative **13a-f** obtained in the previous step, aryl boronic acid **y1-12** (1.1 eq), K_2_CO_3_ (3 eq) and palladium(0) tetrakis(triphenylphosphine) (0.02 eq) were given in this order in a screw‑cap reactor, a 8:8:1 toluene/EtOH/H_2_O mixture (8.5 mL/mmol **13a-f**) was added, the reactor was closed tightly and heated to 100°C under stirring. After 4 h the mixture was cooled to room temperature, diluted with H_2_O (12 mL/mmol **13a-f**), extracted with ethyl acetate (2×25 mL/mmol **13a-f**), the pooled organic phases were washed with 5% NaHCO_3_ (2×12 mL/mmol **13a-f**) and brine (12 mL/mmol **13a-f**), dried over Na_2_SO_4_, filtered and evaporated to dryness under reduced pressure. If necessary, the crude product **(1-12)(a-f)** was purified by preparative HPLC.

*General strategy B*

*Step B1*

To a suspension of 3-boronobenzoic acid in 3:2 dichloromethane/acetonitrile (anhydrous, 5 mL/mmol) in a round-bottomed flask HOBt (1.11 eq) and EDC×HCl (1.10 eq) were added. After complete dissolution the required secondary amine **a-f** (1.2 eq) and DIEA (1.5 eq) were added. After 30 min the mixture was concentrated under reduced pressure, taken-up with AcOEt (40 mL/mmol boronic acid), washed with 2.5% KHSO_4_ (6×10 mL/mmol boronic acid), H_2_O (2×12 mL/mmol boronic acid) and brine (12 mL/mmol boronic acid), dried over Na_2_SO_4_, filtered and evaporated to dryness under reduced pressure. The crude boronic acid intermediate **14a-f** was purified by preparative HPLC.

*Step B2^[2]^*

Aryl bromide **x1-12**, the aryl boronic acid derivative **14a-f** obtained in the previous step (1.05 eq), Na_2_CO_3_ (2 eq) and *N*-tetrabutylammonium bromide (TBAB, 0.01 eq) were given in this order in a screw-cap reactor. H_2_O (2.0 mL/mmol **x1-12**) and 10 mM Pd(EDTA) solution (0.3 mL/mmol aryl **x1-12**) were added, the reactor was closed tightly and heated to 100°C under stirring. After 5 h the mixture was cooled to room temperature, diluted with ethyl acetate (40 mL/mmol **x1-12**), washed with 5 % NaHCO_3_ (15 mL/mmol **x1-12**), H_2_O (15 mL/mmol **x1-12**), 5% KHSO_4_ (15 mL/mmol **x1-12**) and brine (15 mL/mmol **x1-12**), dried over Na_2_SO_4_, filtered and evaporated to dryness under reduced pressure. The crude product was purified by preparative HPLC.

*General strategy C*

*Step C1^[1]^*

3-Bromobenzoic acid, aryl boronic acid **y1-12** (1.1 eq), K_2_CO_3_ (3 eq) and palladium(0) tetrakis(triphenylphosphine) were given in a screw-cap reactor, a 8:8:1 toluene/EtOH/H_2_O mixture (8.5 mL/mmol 3-bromobenzoic acid) was added, the reactor was closed tightly and heated to 100°C under stirring. After 4 h the mixture was cooled to room temperature, diluted with ethyl acetate (10 mL/mmol 3‑bromobenzoic acid) and extracted with 5% NaHCO_3_ (4×10 mL/mmol 3‑bromobenzoic acid). The pooled basic extracts were acidified to pH 3 by dropwise addition of concentrated HCl under stirring, and then extracted with ethyl acetate (3×10 mL/mmol 3‑bromobenzoic acid). The pooled organic extracts were washed with water (10 mL/mmol 3-bromobenzoic acid) and brine (10 mL/mmol 3‑bromobenzoic acid), dried over Na_2_SO_4_, filtered and evaporated to dryness under reduced pressure to yield benzoic acid intermediate **m1-12**.

*Step C2*

To a suspension of the benzoic acid derivative **m1‑12** obtained in step *C1* in anhydrous dichloromethane (5 mL/mmol) in a round-bottomed flask HOBt (1.11 eq) and EDC×HCl (1.10 eq) were added. After complete dissolution secondary amine **a-f** (1.2 eq) and DIEA (1.5 eq) were added. After 30 min the mixture was concentrated under reduced pressure, taken-up with AcOEt (40 mL/mmol **m1-12**), washed with 5% KHSO_4_ (2×15 mL/mmol **m1-12**), H_2_O (12 mL/mmol **m1-12**), 5% NaHCO_3_ (3×12 mL/mmol **m1-12**) and brine (12 mL/mmol benzoic acid), dried over Na_2_SO_4_, filtered and evaporated to dryness under reduced pressure.

*General strategy D*

*Step D1^[1]^*

Aryl bromide **x1-12**, 3-boronobenzoic acid (1.05 eq), Na_2_CO_3_ (2 eq), TBAB (0.01 eq) were given in a screw‑cap reactor. H_2_O (2 mL/mmol **x1-12**) and 10 mM Pd(EDTA) solution (0.3 mL/mmol **x1-12**) were added, the reactor was closed tightly and heated to 100°C under stirring. After 5 h the mixture was cooled to room temperature, diluted with AcOEt (10 mL/mmol **x1-12**) and extracted with 5% NaHCO_3_ (4×10 mL/mmol **x1‑12**). The pooled basic extracts were acidified to pH 3 under stirring by dropwise addition of concentrated HCl and extracted with AcOEt (3×10 mL/mmol **x1-12**). The pooled organic extracts were washed with H_2_O and brine (10 mL/mmol **x1-12** each), dried over Na_2_SO_4_, filtered and evaporated to dryness under reduced pressure. If necessary, the crude benzoic acid intermediate **m1-12** was purified by preparative-HPLC.

*Step D2*

To a suspension of the benzoic acid derivative **m1‑12** obtained in step *C1* in anhydrous dichloromethane (5 mL/mmol) in a round-bottomed flask HOBt (1.11 eq) and EDC×HCl (1.10 eq) were added. After complete dissolution secondary amine **a-f** (1.2 eq) and DIEA (1.5 eq) were added. After 30 min the mixture was concentrated under reduced pressure, taken-up with AcOEt (40 mL/mmol **m1-12**), washed with 5% KHSO_4_ (2×15 mL/mmol **m1-12**), H_2_O (12 mL/mmol **m1-12**), 5% NaHCO_3_ (3×12 mL/mmol **m1‑12**) and brine (12 mL/mmol **m1-12**), dried over Na_2_SO_4_, filtered and evaporated to dryness under reduced pressure.

Synthesis of building blocks and intermediates

Due to the modularity of the general synthesis strategies described above, some intermediates were used for the synthesis of more than one library compound.

*4-Bromophenyl-*tert*-butylcarbonate [****Boc-x7****]*

To a solution of 874 mg 4-bromophenol (5.0 mmol) in 10 mL anhydrous dichloromethane and 0.49 mL pyridine (6.0 mmol) in a round-bottomed flask 12.3 mg 4-(dimethylamino)pyridine (0.1 mmol) and 1.20 g di-*tert*-butyl-dicarbonate (5.3 mmol) were added. After 30 min CO_2_ evolution ceased, the mixture was concentrated under reduced pressure, taken-up in AcOEt (50 mL), washed with 5% KHSO_4_ (2×25 mL), H_2_O (18 mL), 5% Na_2_CO_3_ (2×25 mL) and brine (18 mL), then dried over Na_2_SO_4_, filtered and evaporated to dryness under reduced pressure. 1.25 g 4‑bromophenyl-*tert*-butylcarbonate (Boc-**x7**) were obtained as a white solid (91% yield).

^1^H NMR (CDCl_3_): *δ*/ppm = 7.49 (m, 2H, ArH), 7.07 (m, 2H, ArH), 1.56 (2 s, 9H, Boc CH_3_ cisoid/transoid). ^13^C NMR (CDCl_3_): *δ*/ppm = 27.70, 83.98, 118.77, 123.08, 132.41, 150.14, 151.46.

*4-Bromobenzamide [****x9****]*

In a round-bottomed two-neck flask 812 mg 4‑bromobenzoic acid (4.0 mmol) were suspended in 14 mL of a 6:1 dichloromethane/acetonitrile mixture under argon and 600 mg HOBt (4.44 mmol) and 861 mg EDC×HCl (4.4 mmol) were added under stirring. After 10 min the mixture became clear and was cooled to 0°C in an ice bath. In a round‑bottomed flask 1.64 g NaOH (40 mmol) were added to 2.8 mL of a 28% NH_4_OH solution (20 mmol) and the resulting gaseous NH_3_ was bubbled into the reaction vessel after passing through a NaOH trap. After NH_3_-bubbling had ceased the mixture was concentrated under reduced pressure, taken-up in ethyl acetate (60 mL), washed with 5% KHSO_4_ (2×30 mL), H_2_O (20 mL), 5% NaHCO_3_ (3x20 mL) and brine (20 mL), dried over Na_2_SO_4_, filtered and evaporated to dryness under reduced pressure. 636 mg 4‑bromobenzamide were obtained as an off-white solid (79% yield).

^1^H NMR (*d_6_*-DMSO): *δ*/ppm = 8.04 (br, 1H, cisoid-CONH), 7.81 (m, 2H, ArH), 7.66 (m, 2H, ArH), 7.46 (br, 1H, transoid-CONH). ^13^C NMR (*d_6_*-DMSO): *δ*/ppm = 124.99, 129.58, 131.22, 133.39, 168.87. ESI-MS *m/z*: 200.0 ([M(^79^Br)+H]^+^), 202.2 ([M(^81^Br)+H]^+^, clc 201.97).

*3-Bromobenzamide [****x10****]*

In a round-bottomed two-neck flask 820 mg 3‑bromobenzoic acid (4.0 mmol) were suspended in 14 mL of a 6:1 dichloromethane/acetonitrile mixture under argon and 600 mg HOBt (4.44 mmol) and 861 mg EDC×HCl (4.4 mmol) were added under stirring. After 10 min the mixture became clear and was cooled to 0°C in an ice bath. In a round‑bottomed flask 1.64 g NaOH (40 mmol) were added to 2.8 mL of a 28% NH_4_OH solution (20 mmol) and the resulting gaseous NH_3_ was bubbled into the reaction vessel after passing through a NaOH trap. After NH_3_-bubbling had ceased, the mixture was concentrated under reduced pressure, taken-up in AcOEt (60 mL), washed with 5% KHSO_4_ (2×30 mL), H_2_O (20 mL), 5% NaHCO_3_ (3×20 mL) and brine (20 mL), dried over Na_2_SO_4_, filtered and evaporated to dryness under reduced pressure. 700 mg 3‑bromobenzamide were obtained as an off-white solid (87% yield).

^1^H NMR (*d_6_*-DMSO): *δ*/ppm = 8.08 (br, 1H, cisoid-CONH), 8.04 (t, *J* = 1.8 Hz, 1H, C(2)H), 7.87 (dt, *J* = 7.9, 1.3 Hz, 1H, C(6)H), 7.72 (ddd, *J* = 7.9, 2.0, 1.0, 1H, C(4)H), 7.52 (br, 1H, transoid-CONH), 7.42 (t, J = 7.9 Hz, 1H, C(5)H). ^13^C NMR (*d_6_*-DMSO): *δ*/ppm = 121.61, 126.53, 130.15, 130.50, 133.95, 136.47, 168.28. ESI-MS *m/z*: 200.0 ([M(^79^Br)+H]^+^), 202.2 ([M(^81^Br)+H]^+^).

*4’-Methyl-[1,1’-biphenyl[-3-carboxylic acid [****m1****]*

The reaction was performed according to general procedure D1 using 178 mg 3‑boronobenzoic acid (1.05 mmol) and 126 μL 4‑bromotoluene (1.0 mmol). 186 mg 4’-methyl-[1,1’-biphenyl]-3‑carboxylic acid (***m1***) were obtained as a white solid after preparative HPLC purification (87% yield).

^1^H NMR (CDCl_3_): *δ*/ppm = 8.36 (t, *J* = 1.8 Hz, 1H, C(2)H), 8.10 (dt, *J* = 7.9, 1.4 Hz, 1H, ArH), 7.85 (ddd, *J* = 7.9, 1.9, 1.3, 1H, ArH), 7.55 (m, 3H, ArH), 7.30 (m, 2H, ArH), 2.43 (s, 3H, CH_3_). ^13^C NMR (CDCl_3_): *δ*/ppm = 21.17, 127.03, 128.67, 128.70, 128.97, 129.69, 129.78, 132.25, 137.07, 137.75, 141.59, 171.98. ESI-MS *m/z*: 254.3 ([M+H+MeCN]^+^), 425.2 ([2M+H]^+^).

*3-(6’-Methylpyridin-3’-yl)-benzoic acid hydrochloride [****m2****×HCl]*

The reaction was performed according to general procedure D1 using 178 mg 3-boronobenzoic acid (1.05 mmol) and 176 mg 5‑bromo‑2-methylpyridine (1.0 mmol). After acidification of the basic extracts, the aqueous phase was saturated with NaCl and extracted with 1-butanol (4×10 mL). The pooled organic extracts were washed with brine (10 mL), dried over Na_2_SO_4_, filtered, concentrated under reduced pressure, taken-up with toluene (5 mL) and evaporated to dryness under reduced pressure. 222 mg 3-(6’-methylpyridin-3’-yl)-benzoic acid hydrochloride (**m2***×*HCl) were obtained as a white solid (85% yield).

^1^H NMR (CDCl_3_): *δ*/ppm = 9.11 (d, J = 2.1 Hz, 1H, C(2’)H), 8.73 (dd, J = 8.3, 2.1 Hz, 1H, C(4’)H), 8.33 (t, J = 1.6 Hz, 1H, C(2)H), 8.10 (ddd, *J* = 8.0, 1.8, 1.2 Hz, 1H, ArH), 8.06 (dt, *J* = 7.7, 1.2 Hz, 1H, ArH), 7.92 (d, J = 8.3 Hz, 1H, C(5’)H), 7.70 (t, J = 7.7 Hz, C(5)H), 2.77 (s, 3H, CH_3_). ^13^C NMR (CDCl_3_): *δ*/ppm = 19.41, 127.33, 127.88, 129.69, 129.96, 131.58, 131.90, 134.53, 134.93, 137.50, 139.90, 142.23, 153.18, 166.83. ESI-MS *m/z*: 214.1 ([M+H]^+^).

*3’-Fluoro-4’-methyl-[1,1’-biphenyl]-3-carboxylic acid [****m3****]*

The reaction was performed according to general procedure D1 using 178 mg 3-boronobenzoic acid (1.05 mmol) and 129 μL 4‑bromo‑2-fluorotoluene (1.0 mmol). 205 mg 3’-fluoro-4’-methyl-[1,1’-biphenyl]-3-carboxylic acid (**m3**) were obtained as a white solid after preparative HPLC purification (87% yield).

^1^H NMR (CDCl_3_): *δ*/ppm = 8.33 (t, *J* = 1.7 Hz, C(2)H), 8.11 (dt, J = 7.7, 1.3 Hz, 1H, C(6)H), 7.82 (m, 1H, C(4)H), 7.56 (t, J = 7.7 Hz, 1H, C(5)H), 7.32 (m, 3H, ArH), 2.34 (d, *J*_HF_ = 1.6 Hz, 3H, CH_3_). ^13^C NMR (CDCl_3_): *δ*/ppm = 14.35 (*J*_HF_ = 3.8 Hz), 113.64 (*J*_HF_ = 22.5 Hz), 122.42 (J_HF_ = 3.8 Hz), 124.63 (J_HF_ = 13.5 Hz), 128.61, 129.12, 129.91, 129.84, 131.94 (*J*_HF_ = 6.0 Hz), 132.15, 171.23. ESI-MS *m/z*: 272.2 ([M+H+MeCN]^+^), 461.2 ([2M+H]^+^).

*4’-Fluoro-[1,1’-biphenyl]-3-carboxylic acid [****m4****]*

The reaction was performed according to general procedure D1 using 178 mg 3-boronobenzoic acid (1.05 mmol) and 111 μL 1‑bromo‑4‑fluorobenzene (1.0 mmol). 190 mg 4’-fluoro-[1,1’‑biphenyl]-3-carboxylic acid (**m4**) were obtained as a white solid (86% yield).

^1^H NMR (*d_6_*-DMSO): *δ*/ppm = 13.07 (br, 1H, COOH), 8.16 (t, *J* = 1.6 Hz, 1H, C(2)H), 7.91 (m, 2H, ArH), 7.74 (m, 2, ArH), 7.59 (t, J = 7.9 Hz, C(5)H), 7.32 (m, 2H, ArH).

*4’-Chloro-[1,1’-biphenyl]-3-carboxylic acid [****m5****]*

The reaction was performed according to general procedure D1 using 178 mg 3-boronobenzoic acid (1.05 mmol) and 193 mg 1-bromo-4-chlorobenzene (1.0 mmol). 94 mg 4’‑chloro-[1,1’‑biphenyl]-3-carboxylic acid (**m5**) were obtained as a white solid after preparative HPLC purification (39% yield).

^1^H NMR (CDCl_3_): *δ*/ppm = 8.26 (t, *J* = 1.6 Hz, C(2)H), 8.05 (dt, *J* = 7.7, 1.4 Hz, 1H, C(4)H), 7.76 (ddd, J = 7.9, 1.9, 1.2 Hz, 1H, C(6)H), 7.54 (m, 3H, ArH), 7.42 (m, 2H, ArH). ^13^C NMR (CDCl_3_): *δ*/ppm = 125.90, 125.93, 126.49, 126.52, 126.54, 128.20, 129.03 131.40, 136.04, 137.72, 166.41.

*2’-Chloro-[1,1’-biphenyl]-3-carboxylic acid [****m6****]*

The reaction was performed according to general procedure D1 using 356 mg 3-boronobenzoic acid (2.1 mmol) and 236 μL 1-bromo-2‑chlorobenzene (2.0 mmol). 203 mg 2’-chloro-[1,1’‑biphenyl]-3-carboxylic acid (**m6**) were obtained as a white solid after preparative HPLC purification (44% yield).

^1^H NMR (CDCl_3_): *δ*/ppm = 8.21 (t, *J* = 1.5 Hz, 1H, C(2)H), 8.15 (dt, *J* = 7.8, 1.4 Hz, 1H, C(4)H), 7.73 (dt, *J* = 7.8, 1.5 Hz, 1H, C(6)H), 7.56 (t, *J* = 7.8 Hz, 1H, C(5)H), 7.51 (m, 1H, ArH), 7.34 (m, 3H, ArH). ^13^C NMR (CDCl_3_): *δ*/ppm = 127.02, 128.31, 129.10, 129.41, 130.08, 131.22, 131.31, 132.51, 134.89, 139.32, 139.52, 171.33. ESI-MS *m/z*: 215.2 ([M(^35^Cl)-OH]^+^), 467.1 ([M(^35^Cl)+M(^37^Cl)+H]^+^).

*4-*tert*-butyloxycarbonyloxy-[1,1’-biphenyl]-3-carboxylic acid [Boc****-m7****]*

276 mg **Boc-x7** (1.0 mmol), 186 mg 3-boronobenzoic acid (1.05 mmol), 419 mg K_2_CO_3_ (3.0 mmol) and 23 mg palladium(0) tetrakis(triphenylphosphine) (0.02 mmol) were given in a screw-cap reactor, 4.5 mL of a 8:8:1 toluene/EtOH/H_2_O mixture were added, the reactor was closed tightly and heated to 100°C under stirring. After 4 h the mixture was cooled to room temperature, diluted with AcOEt (10 mL) and extracted with 3% Na_2_CO_3_ (4×15 mL). The pooled basic extracts were acidified to pH 3 by dropwise addition of 6 *N* HCl under stirring, whereby a solid precipitated, which was filtered and washed with 6 *N* HCl and H_2_O. The crude product was dissolved in AcOEt (30 mL), washed with H_2_O (10 mL) and brine (10 mL), then dried over Na_2_SO_4_, filtered and evaporated to dryness under reduced pressure. 150 mg 4-tert-butyloxycarbonyloxy-[1,1’-biphenyl]-3-carboxylic acid (Boc-**m7**) were obtained as a white solid (46% yield).

^1^H NMR (CDCl_3_): *δ*/ppm = 8.31 (t, *J* = 1.4 Hz, Ar C(2)H), 8.09 (m, 1H, ArH), 7.79 (dt, *J* = 8.3, 1.6 Hz, 1H, ArH), 7.62 (m, 2H, ArH), 7.54 (t, *J* = 7.7 Hz, Ar C(5)H), 7.27 (m, 2H, ArH), 1.58 (s, 9H, Boc CH_3_). ^13^C NMR (CDCl_3_): *δ*/ppm = 27.78, 83.79, 121.78, 128.21, 128.79, 129.06 (br), 132.26, 137.63, 140.76, 150.38, 150.94, 151.84, 171.60. ESI-MS *m/z*: 300.1 ([M‑*t*Bu+2H+MeCN]^+^), 629.1 ([2M+H]^+^, clc 629.25).

*(4’-*tert*-butyloxycarbonyloxy-[1,1’-biphenyl]-3-yl)(4-methylpiperidin-1-yl)methanone [Boc****-7b****]*

The reaction was performed according to general procedure D2 using 142 mg Boc-**m7** (0.44 mmol) and 65 μL 4-methylpiperidine (0.53 mmol). 180 mg title compound Boc-**7b** were obtained as an oil (quantitative yield).

^1^H NMR (CDCl_3_): *δ*/ppm = 7.58 (m, 4H, ArH), 7.45 (m, 1H, ArH), 7.36 (dt, *J* = 7.5, 1.3 Hz, 1H, ArH), 7.25 (m, 2H, ArH), 4.70 (br, 1H, piperidine CH), 3.75 (br, 1H, piperidine CH), 3.03 (br, 1H, piperidine CH), 2.79 (br, 1H, piperidine CH), 1.79 (br, 1H, piperidine C(4)H), 1.64 (m, 2H, piperidine CH), 1.58 (s, 9H, Boc CH_3_), 1.24 (m, 2H, piperidine CH), 0.98 (d, *J* = 6.4 Hz, 3H, piperidine CH_3_). ^13^C NMR (CDCl_3_): *δ*/ppm = 21.76, 27.75, 31.22, 83.76, 121.71, 125.48, 125.71, 128.05, 128.17, 128.96, 137.17, 138.12, 140.62, 150.79, 151.58, 170.10. ESI-MS *m/z*: 396.2 ([M+H]^+^.

*3’-Hydroxy-[1,1’-biphenyl]-3-carboxylic acid [****m8****]*

The reaction was performed according to general procedure D1 using 178 mg 3-boronobenzoic acid (1.05 mmol) and 112 μL 3‑bromophenol (1.0 mmol). 183 mg 3’-hydroxy-[1,1’-biphenyl]-3-carboxylic acid (**m8**) were obtained as a pale orange sold (84% yield).

^1^H NMR (*d_6_*-DMSO): *δ*/ppm = 13.05 (br, 1H, COOH), 9.57 (br, 1H, OH), 8.11 (t, *J* = 1.6 Hz, Ar C(2)H), 7.92 (dt, *J* = 7.9, 1.4 Hz, 1H, ArH), 7.85 (ddd, *J* = 7.8, 1.9, 1.3 Hz, 1H, ArH), 7.57 (t, *J* = 7.8 Hz, 1H, ArH), 7.28 (t, *J* = 7.9 Hz, 1H, ArH), 7.10 (ddd, *J* = 7.5, 1.5, 1.1 Hz, 1H, ArH), 7.05 (t, J = 2.0 Hz, 1H, Ar C(2’)H), 6.80 (ddd, *J* = 8.1, 2.4, 0.9 Hz, 1H, ArH). ESI-MS *m/z*: 256.4 ([M+H+MeCN]^+^), 429.1 ([2M+H]^+^, clc 429.13).

*3-carboxy-[1,1’-biphenyl]-4’-carboxamide [****m9****]*

The reaction was performed according to general procedure D1 using 178 mg 3-boronobenzoic acid (1.05 mmol) and 202 mg **x9** (1.00 mmol). 227 mg 3-carboxy-[1,1’-biphenyl]-4’-carboxamide (**m9**) were obtained as a white solid which precipitated from the reaction mixture after acidification to pH 3 (93% yield).

^1^H NMR (*d_6_*-DMSO): *δ*/ppm = 13.13 (br, 1H, COOH), 8.23 (br, 1H ArH), 8.02 (m, 5H, 1 CONH and 4 ArH), 7.80 (m, 2H, ArH), 7.62 (t, *J* = 7.7 Hz, 1H, Ar C(5)H), 7.40 (br, 1H, CONH’). ^13^C NMR (*d_6_*-DMSO): *δ*/ppm = 126.58, 127.43, 128.29, 128.74, 129.41, 131.27, 131.59, 133.51, 139.58, 141.77, 167.10, 167.41. ESI-MS *m/z*: 242.2 ([M+H]^+^).

*3-carboxy-[1,1’-biphenyl]-3’-carboxamide [****m10****]*

The reaction was performed according to general procedure D1 using 178 mg 3-boronobenzoic acid (1.05 mmol) and 201 mg **x10** (1.00 mmol). 221 mg 3‑carboxy-[1,1’-biphenyl]-3’‑carboxyamide (**m10**) were obtained as a white solid which precipitated from the reaction mixture after acidification to pH 3 (90% yield).

^1^H NMR (*d_6_*-DMSO): *δ*/ppm = 13.12 (br, 1H, COOH), 8.26 (s, 1H, ArH), 8.20 (s, 1H, ArH), 8.16 (br, 1H, CONH), 7.98 (m, 2H, ArH), 7.88 (m, 2H, ArH), 7.63 (t, *J* = 7.9 Hz, 1H, ArH), 7.58 (t, *J* = 7.8 Hz, 1H, ArH), 7.45 (br, 1H, CONH’). ^13^C NMR (*d_6_*-DMSO): *δ*/ppm = 125.71, 127.04, 127.45, 128.53, 129.10, 129.37, 129.51, 131.25, 131.56, 135.05, 139.19, 139.90, 167.18, 167.63. ESI-MS *m/z*: 242.2 ([M+H]^+^).

*N-(3-bromobenzoyl)-azepane [****13e****]*

The reaction was performed according to general procedure A1 using 410 mg 3-bromobenzoic acid (2.0 mmol) and 275 μL azepane (2.4 mmol). 545 mg *N*-(3-bromobenzoyl)-azepane were obtained as an oil (96% yield).

^1^H NMR (CDCl_3_): *δ*/ppm = 7.52 (m, 2H, ArH), 7.28 (m, 2H, ArH), 3.66 (m, 2H, azepane CH_2_), 3.35 (m, 2H, azepane CH_2_), 1.83 (m, 2H, azepane CH_2_), 1.63 (m, 6H, 3 azepane CH_2_). ^13^C NMR (CDCl_3_): *δ*/ppm = 26.49, 27.23, 27.84, 29.84, 46.40, 49.78, 122.54, 125.04, 129.57, 130.05, 132.15, 139.29, 169.82. ESI-MS *m/z*: 282.2 ([M(^79^Br)+H]^+^), 284.2 ([M(^81^Br)+H]^+^).

*N-(3-boronobenzoyl)-4-methylpiperidine [****14b****]*

The reaction was performed according to general procedure B1 using 169 mg 3-boronobenzoic acid (1.0 mmol) and 148 μL 4‑methylpiperidine (1.2 mmol). 108 mg *N*-(3-boronobenzoyl)-4‑methylpiperidine were obtained as an oil (42% yield).

^1^H NMR (CDCl_3_/*d_6_*-DMSO 1:1): *δ*/ppm = 7.83 (m, 2H, ArH), 7.39 (m, 2H, ArH), 6.90 (br, 2H, B(OH)_2_), 4.62 (br, 1H, piperidine CH), 3.64 (br, 1H, piperidine CH), 2.95 (m, 1H, piperidine CH), 2.58 (m, 1H, piperidine CH), 1.73 (m, 1H, piperidine C(4)H), 1.58 (m, 2H, piperidine CH), 1.11 (m, 2H, piperidine CH), 0.94 (d, *J* = 6.2 Hz, 3H, piperidine CH_3_). ^13^C NMR (CDCl_3_): *δ*/ppm = 26.49, 27.23, 29.69, 31.09, 33.88, 34.62, 127.54, 128.60, 132.36, 135.21, 135.49, 170.72. ESI-MS *m/z*: 247.3 ([M(^10^B)+H]^+^), 248.2 ([M(^11^B)+H]^+^).

*N-(3-boronobenzoyl)-azepane [****14e****]*

The reaction was performed according to general procedure B1 using 1.016 g 3-boronobenzoic acid (6.0 mmol) and 0.82 mL azepane (7.2 mmol). 1.179 g *N*-(3-boronobenzoyl)-azepane were obtained as a white solid after preparative HPLC purification (79% yield).

^1^H NMR (CDCl_3_): *δ*/ppm = 8.10 (br, 1H, B-OH), 7.81 (m, 1H, ArH), 7.75 (s, 1H, ArH), 7.40 (m, 2H, ArH), 4.36 (m, 1H, B-OH’), 3.56 (t, *J* = 6.3 Hz, 2H, azepane CH_2_), 3.29 (m, 2H, azepane CH_2_), 1.74 (m, 2H, azepane CH_2_), 1.52 (m, 6H, 3 azepane CH_2_). ^13^C NMR (CDCl_3_): *δ*/ppm = 25.82, 26.83, 27.17, 27.85, 45.30, 49.13, 127.32, 127.76, 131.74, 134.49, 136.47, 170.64. ESI‑MS *m/z*: 247.3 ([M(^10^B)+H]^+^), 248.2 ([M(^11^B)+H]^+^).

Synthesis of the library compounds

*(4’-Methyl-[1,1’-biphenyl]-3-yl)(4-methylpiperidin-1-yl)methanone [****1b****]*

The reaction was performed according to general strategy D2 using 128 mg **m1** (0.60 mmol) and 89 μL 4‑methylpiperidine ( 0.72 mmol). 154 mg title compound **1b** were obtained as an oil (87% yield).

^1^H NMR (CDCl_3_): *δ*/ppm = 7.62 (m, 2H, ArH), 7.50 (m, 2H, ArH), 7.45 (m, 1H, ArH), 7.34 (m, 1H, ArH), 7.26 (m, 1H, ArH), 4.70 (br, 1H, piperidine CH), 3.78 (br, 1H, piperidine CH), 2.99 (br, 1H, piperidine CH), 2.80 (br, 1H, piperidine CH), 2.41 (s, 3H ArCH_3_), 1.58-1.76 (m, 3H, piperidine CH), 0.99 (d, *J* = 6.2 Hz, RCH_3_). ^13^C NMR (CDCl_3_): *δ*/ppm = 21.14, 21.78, 31.23, 33.90, 34.78, 42.59, 48.15, 125.31, 126.99, 127.88, 128.03, 129.60, 137.05, 137.49, 137.57, 141.39, 170.30. ESI-MS *m/z*: 294.3 ([M+H]^+^). HR-MS *m*/*z*: 294.1856 ([M+H]^+^, C_20_H_24_NO requires 294.1852).

*Azepan-1-yl(4’-methyl-[1,1’-biphenyl]-3-yl)methanone [****1e****]*

The reaction was performed according to general strategy D2 using 185 mg **m1** (0.87 mmol) and 100 μL azepane (0.90 mmol). 250 mg title compound **1e** were obtained as a pale yellow solid (97% yield).

Mp: 109-111°C. ^1^H NMR (CDCl_3_): *δ*/ppm = 7.60 (m, 2H, ArH), 7.50 (m, 2H, ArH), 7.45 (td, *J* = 7.4, 1.0 Hz, 1H, CH Ar), 7.32 (m, 1H, ArH), 7.27 (m, 2H, ArH), 3.70 (t, *J* = 5.8 Hz, 2H, azepane CH), 3.42 (m, 2H, azepane CH), 2.41 (s, 3H, ArCH_3_), 1.87 (m, 2H, azepane CH), 1.62 (m, 6H, azepane CH). ^13^C NMR (CDCl_3_): *δ*/ppm = 21.14, 26.54, 27.33, 27.90, 29.62, 46.38, 49.83, 124.98, 126.99, 127.57, 128.82, 129.59, 137.47, 137.61, 137.88, 141.34, 171.57. ESI-MS *m/z*: 294.3 ([M+H]^+^). HR-MS *m*/*z*: 294.1857 ([M+H]^+^, C_20_H_23_NO requires 294.1852).

*(4-Methylpiperidin-1-yl)(3-(6-methylpyridin-3-yl)phenyl)methanone [****2b****]*

To a suspension of ***m2****×HCl* (0.81 mmol) in a 2:1 dichloromethane/aceetonitrile mixture (6 mL) in a round-bottomed flask 0.15 mL DIEA (0.89 mmol) were added. After 5 min 186 mg HOBt (1.37 mmol) and 264 mg EDC×HCl (1.35 mmol) were added under stirring. After 30 min 147 μL 4‑methylpiperidine (0.98 mmol) and 0.21 mL DIEA (1.24 mmol) were added. After 50 min the mixture was concentrated under reduced pressure, taken-up with AcOEt (45 mL), washed with 5% NaHCO_3_ (4×15 mL) and H_2_O (15 mL) and extracted with 5% KHSO_4_ (2×22 mL). The pooled acidic extracts were brought to pH 8 by addition of solid Na_2_CO_3_ and extracted with AcOEt (4×15 mL), the pooled organic extracts were washed with H_2_O (10 mL) and brine (10 mL), dried over Na_2_SO_4_, filtered and evaporated to dryness under reduced pressure. 181 mg title compound **2b** were obtained as a waxy solid (74% yield).

^1^H NMR (CDCl_3_): *δ*/ppm = 8.72 (d, *J* = 1.9 Hz, 1H, pyH), 7.79 (dd, *J* = 7.9, 1.4 Hz, 1H, pyH), 7.60 (m, 2H, ArH), 7.49 (m, 1H, ArH), 7.39 (dt, *J* = 7.7, 1.4 Hz, 1H, ArH), 7.25 (d, *J* = 8.1 Hz, 1H, pyH), 4.70 (br m, 1H, piperidine CH), 3.75 (br m, 1H, piperidine CH), 3.02 (br m, 1H, piperidine CH), 2.81 (br m, 1H, piperidine CH), 2.62 (s, 3H pyCH_3_), 1.76 (br m, 1H, piperidine CH), 1.62 (m, 2H, piperidine CH), 1.23 (br m, 3H, piperidine CH), 0.98 (d, *J* = 6.4, 3H, piperidine CH_3_). ^13^C NMR (CDCl_3_): *δ*/ppm = 21.75, 24.08, 31.20, 33.81 (br), 34.72 (br), 42.60 (br), 48.19 (br), 123.34, 125.41, 126.11, 127.89, 129.15, 133.09, 134.88, 137.43, 138.24, 147.36, 157.62, 169.88. ESI-MS *m/z*: 295.2 ([M+H]^+^). HR-MS *m*/*z*: 295.1813 ([M+H]^+^, C_19_H_22_N_2_O requires 295.1805).

*Azepan-1-yl-(3-(6-methylpyridin-3-yl)phenyl)methanone [****2e****]*

The reaction was performed according to general strategy B2 using 262 mg **14e** (1.05 mmol) and 175 mg 5‑bromo-2-methylpyridine (1.0 mmol). 150 mg title compound **2e** were obtained as an oil after preparative HPLC purification (50% yield).

^1^H NMR (CDCl_3_): *δ*/ppm = 8.73 (d, *J* = 2.4 Hz, 1H, pyH), 7.79 (dd, *J* = 8.1, 2.4 Hz, 1H, pyH), 7.59 (m, 2H, ArH), 7.52 (m, 1H, ArH), 7.38 (m, 1H, ArH), 7.24 (d, *J* = 8.1 Hz, 1H, pyH), 3.71 (t, *J* = 5.8 Hz, 2H, azepane CH) 3.41 (m, 2H, azepane CH), 2.62 (s, 3H pyCH_3_), 1.88 (m, 2H, azepane CH), 1.62 (m, 6H, azepane CH). ^13^C NMR (CDCl_3_): *δ*/ppm = 24.08, 26.53, 29.63, 46.43, 49.83, 123.32, 125.04, 125.80, 127.59, 129.15, 133.14, 134.89, 138.18, 138.27, 147.37, 157.60, 171.17. ESI-MS *m/z*: 295.2 ([M+H]^+^). HR‑MS *m*/*z*: 295.1810 ([M+H]^+^, C_19_H_22_N_2_O requires 295.1805).

*(3,3-Dimethylpiperidin-1-yl)(3’-fluoro-4’-methyl-[1,1’-biphenyl]-3-yl)methanone [****3a****]*

The reaction was performed according to general strategy D2 using 165 mg **m3** (0.68 mmol) and 98 μL 3,3-dimethylpiperidine (0.67 mmol). 208 mg title compound **3a** were obtained as an oil (94% yield).

^1^H NMR (CDCl_3_): *δ*/ppm = 7.58 (m, 2H, ArH), 7.46 (t, *J* = 7.8 Hz, 1H, ArH), 7.35 (m, 1H, ArH), 7.27 (m, 3H, ArH), 3.71 (br, 1H, piperidine CH), 3.46 (br, 1H, piperidine CH), 3.34 (br, 1H, piperidine CH), 3.07 (br, 1H, piperidine CH), 2.32 (d, *J*_HF_ = 1.9 Hz, 3H, ArCH_3_), 1.70 (br, 1H, piperidine CH), 1.59 (br, 1H, piperidine CH), 1.46 (m, 2H, piperidine C(4)H_2_), 1.04 (br, 3H, piperidine CH_3_), 0.85 (br, 3H, piperidine C’H_3_). ^13^C NMR (CDCl_3_)[[1]](#footnote-2): *δ*/ppm = 21.31, 28.06 (br), 37.96 (br), 42.82/42.89, 53.32/53.41, 113.58 (d, *J*_HF_ = 23.2 Hz), 122.36 (d, *J*_HF_ = 3.0 Hz), 124.18 (d, *J*_HF_ = 17.2 Hz), 125.24/125.38, 125.71/126.09, 127.69/127.72, 128.95, 131.85 (d, *J*_HF_ = 6.0 Hz), 137.29/137.33, 139.90 (d, *J*_HF_ = 6.0 Hz)/139.95 (d, *J*_HF_ = 8.2 Hz), 145.98, 162.22 (d, *J*_HF_ = 155.1 Hz), 166.16/170.76. ESI-MS *m/z*: 326.2 ([M+H]^+^). HR-MS *m*/*z*: 326.1918 ([M+H]^+^, C_21_H_25_FNO requires 326.1915).

*(4’-fluoro-3’-methyl-[1,1’-biphenyl]-3-yl)(4-methylpiperidin-1-yl)methanone [****3b****]*

101 mg **14b** (0.41 mmol), 50 μL 4‑bromo-3-fluorotoluene (0.39 mmol), 162 mg K_2_CO_3_ (1.16 mmol) and palladium(0) tetrakis(triphenylphosphine) (7.7 μmol) were given in a screw-cap reactor, 3.4 mL of a 8:8:1 mixture of toluene/EtOH/H_2_O were added, the reactor was closed tightly and heated to 100°C under stirring. After 3 h the mixture was cooled to room temperature, diluted with H_2_O (5 mL) and extracted with ethyl acetate (2×10 mL), the pooled organic phases were washed with 5% NaHCO_3_ (10 mL) and brine (5 mL), dried over Na_2_SO_4_, filtered and evaporated to dryness under reduced pressure. The crude product was purified by preparative-HPLC, yielding 89 mg of the title compound **3b** as an oil (74% yield).

^1^H NMR (CDCl_3_): *δ*/ppm = 7.60 (m, 2H, ArH), 7.46 (m, 1H, ArH), 7.37 (m, 1H, ArH), 7.26 (m, 3H, ArH), 4.71 (br, 1H, piperidine CH), 3.76 (br, 1H, piperidine CH), 2.94 (br, 1H, piperidine CH), 2.80 (br, 1H, piperidine CH), 2.32 (d, *J*_HF_ = 1.6, 3H, ArCH_3_), 1.68 (m, 3H, piperidine H), 1.25 (m, 2H, piperidine CH), 0.99 (d, *J* = 6.2, piperidine CH_3_). ^13^C NMR (CDCl_3_): *δ*/ppm = 14.33 (d, *J*_HF_ = 3.0), 21.73, 31.22, 33.97, 34.86, 42.58, 48.13, 113.59 (d, *J*_HF_ = 24.0), 122.37 (d, J_HF_ = 3.0), 124.18 (d, *J*_HF_ = 17.2), 125.31, 125.83, 127.82, 128.95, 131.85 (d, *J*_HF_ = 6.0), 137.22, 139.95 (d, *J*_HF_ = 8.2), 140.28 (d, *J*_HF_ = 2.2), 161.63 (d, *J*_HF_ = 243), 170.06. ESI-MS *m/z*: 312.3 ([M+H]^+^). HR-MS *m*/*z*: 312.1764 ([M+H]^+^, C_20_H_23_FNO requires 312.1758).

*(2,2-Dimethylmorpholino)(3’-fluoro-4’-methyl-[1,1’-biphenyl]-3-yl)methanone [****3c****]*

The reaction was performed according to general strategy D2 using 178 mg **m3** (0.76 mmol) and 91 mg 2,2‑dimethylmorpholine (0.75 mmol). 219 mg title compound **3c** were obtained as an oil (88% yield).

^1^H NMR (CDCl_3_): *δ*/ppm = 7.62 (m, 2H, ArH), 7.49 (t, *J* = 7.7 Hz, 1H, ArH), 7.36 (br, 1H, ArH), 7.26 (m, 3H, ArH), 3.69 (br, 4H, morpholine CH), 3.44 (br, 1H, morpholine CH), 3.26 (m, 1H, morpholine CH), 3.44 (m, 1H, morpholine CH), 2.32 (d, *J*_HF_ = 1.8, 3H, ArCH_3_), 1.31 (br, 3H, morpholine CH_3_), 1.18 (br, 3H, morpholine C’H_3_). ^13^C NMR (CDCl_3_)^a^: *δ*/ppm = 14.34 (d, *J*_HF_ = 3.7 Hz), 24.17 (br), 40.11/40.25, 51.17/51.30, 60.25/60.37, 113.60 (d, *J*_HF_ = 23.0 Hz), 122.36 (d, *J*_HF_ = 3.0 Hz), 124.37 (d, *J*_HF_ = 17.2 Hz), 125.49, 125.97, 128.22, 129.12, 131.91 (d, *J*_HF_ = 5.2 Hz), 136.23, 139.70 (d, *J*_HF_ = 7.5 Hz), 140.53, 161.63 (d, *J*_HF_ = 243 Hz), 167.27. ESI‑MS *m/z*: 328.2 ([M+H]^+^). HR-MS *m*/*z*: 328.1713 ([M+H]^+^, C_20_H_23_NO_2_ requires 328.1707).

*(2,6-Dimethylmorpholino)(3’-fluoro-4’-methyl-[1,1’-biphenyl]-3-yl)methanone [****3d****]*

The reaction was performed according to general procedure D2 using 183 mg **m3** (0.79 mmol) and 121 μL 2,6-dimethylmorpholine (0.95 mmol). 251 mg title compound **3d** were obtained as as an oil (95% yield). Due to the composition of the secondary amine used, the product was obtained as a mixture of two diastereomeric pairs of enantiomers in about 4:1 ratio.

^1^H NMR (CDCl_3_): *δ*/ppm = 7.62 (m, 2H, ArH), 7.49 (m, 1H, ArH), 7.36 (m, 1H, ArH), 7.26 (m, 3H, ArH), 4.61 (br, 1H, morpholine CH major diastereomer), 3.97 (br, 2H, morpholine CH minor diastereomer), 3.62 (br m, 3H, morpholine CH both diastereomers), 3.21 (br, 1H, morpholine CH minor diastereomer) 2.83 (br, 1H, morpholine CH major diastereomer), 2.57 (br, 1H, morpholine CH major diastereomer), 2.33 (m, 3H, ArCH_3_ both diastereomers), 1.27 (br, 3H, morpholine CH_3_), 1.12 (br, 3H, morpholine C’H_3_). ^13^C NMR (CDCl_3_, signals of the major diastereomer only): *δ*/ppm = 14.34 (d, *J*_HF_ = 3.0 Hz), 18.66, 18.68, 72.02, 113.60 (d, *J*_HF_ = 23.2 Hz), 122.36 (d, *J*_HF_ = 3.0 Hz), 124.25, 125.61, 126.01, 128.26, 129.08, 131.90 (d, *J*_HF_ = 5.2 Hz), 139.71 (d, *J*_HF_ = 7.5 Hz), 140.52, 161.64 (d, *J*_HF_ = 244 Hz), 169.91. ESI-MS minor diastereomer *m/z*: 328.2 ([M+H]^+^); major diastereomer ([M+H]^+^). HR-MS minor diastereomer *m*/*z*: 328.1711 ([M+H]^+^, C_20_H_23_NO_2_ requires 328.1707); major diastereomer *m*/*z*: 328.1713 ([M+H]^+^, C_20_H_23_NO_2_ requires 328.1707).

*Azepan-1-yl(3’-fluoro-4’-methyl-[1,1’-biphenyl]-3-yl)methanone [****3e****]*

The reaction was performed according to general strategy A2 applying a different literature cross-coupling protocol [3]: 264 mg **13e** (0.92 mmol), 217 mg 3‑fluoro-4-tolylboronic acid (1.36 mmol), 194 mg Na_2_CO_3_ (1.84 mmol), 10.4 mg palladium acetate (0.046 mmol), 29.5 mg tris(2‑methylphenyl)-phosphine (0.092 mmol) were given in a screw-cap reactor, 1,2‑dimethoxyethane (5.5 mL) and H_2_O (1.0 mL) were added, the reactor was closed tightly and heated to 80°C under stirring. After 4 h the mixture was cooled to room temperature, diluted with H_2_O (10 mL) and extracted with AcOEt (2x20 mL), the pooled organic extracts were washed with 5% NaHCO_3_ (2x10 mL) and brine (10 mL), dried over Na_2_SO_4_, filtered and evaporated to dryness under reduced pressure. The crude product was purified by preparative HPLC. 205 mg of the title compound **3e** were obtained as an off-white solid after HPLC purification (71% yield).

Mp: 106-108°C. ^1^H NMR (CDCl_3_): *δ*/ppm = 7.58 (m, 2H, ArH), 7.45 (m, 1H, ArH), 7.35 (m, 1H, ArH), 7.26 (m, 3H, ArH), 3.71 (t, *J* = 5.8 Hz, 2H, azepane CH), 3.41, (m, 2H, azepane CH), 2.32 (d, *J*_HF_ = 1.8 Hz, 3H, ArCH_3_), 1.87 (m, 2H, azepane CH), 1.64 (m, 6H, azepane CH). ^13^C NMR (CDCl_3_): *δ*/ppm = 14.32 (d, J_HF_ = 3.8 Hz), 26.53, 27.31, 27.90, 29.62, 46.38, 49.83, 113.60 (d, *J*_HF_ = 23.2 Hz), 122.37 (d, *J*_HF_ = 3.0 Hz), 124.97, 125.51, 127.50, 128.94, 131.83 (d, *J*_HF_ = 6.0 Hz), 138.04, 139.99 (d, *J*_HF_ = 7.5 Hz), 140.22 (d, *J*_HF_ = 2.2 Hz), 161.63 (d, *J*_HF_ = 240 Hz), 171.34. ESI-MS *m/z*: 312.3 ([M+H]^+^). HR-MS *m*/*z*: 312.1764 ([M+H]^+^, C_20_H_23_FNO requires 312.1758).

*(*S*)-1-(3’-fluoro-4’-methyl-[1,1’-biphenyl]-3-carbonyl)-pyrrolidine-2-carboxamide [****3f****]*

The reaction was performed according to general procedure D2 using 248 mg **m3** (1.00 mmol) and 228 mg TFA×H-(*S*)-Pro-NH_2_ (1.00 mmol). 220 mg title compound **3f** were obtained a white solid (97% yield).

Mp: 73-75°C. ^1^H NMR (CDCl_3_): *δ*/ppm = 7.71 (m, 1H, ArH), 7.64 (m, 1H, ArH), 7.49 (m, 2H, ArH), 7.26 (m, 3H, ArH), 7.00 (br, 1H, CONH), 5.52 (br, 1H, CONH’), 4.83 (dd, *J* = 7.5, 1.9 Hz, 1H Pro αCH), 3.58 (m, 2H, Pro δCH_2_), 2.49 (m, 1H, Pro βCH), 2.32 (d, *J* = 1.7 Hz, 3H, ArCH_3_), 2.10 (m, 2H, Pro CH), 1.88 (m, 1H, Pro CH). ^13^C NMR (CDCl_3_): *δ*/ppm = 14.32 (d, *J*_HF_ = 2.2 Hz), 25.46, 27.49, 50.58, 59.64, 113.59 (d, *J*_HF_ = 23.2 Hz), 122.38 (d, *J*_HF_ = 2.2 Hz), 124.37 (d, *J*_HF_ = 17.2 Hz), 125.65, 126.13, 128.77, 128.98, 131.90 (d, *J*_HF_ = 5.2 Hz), 136.76, 139.64 (d, *J*_HF_ = 6.8 Hz), 140.32, 164.07 (d, *J*_HF_ = 126 Hz), 170.90, 173.45. ESI-MS *m/z*: 327.2 ([M+H]^+^). HR-MS *m*/*z*: 327.1505 ([M+H]^+^, C_19_H_20_FN_2_O_2_ requires 327.1503).

*(4’-Fluoro-[1,1’-biphenyl]-3-yl)(4-methylpiperidin-1-yl)methanone [****4b****]*

The reaction was performed according to general procedure D2 using 180 mg **m4** (0.83 mmol) and 123 μL 4‑methylpiperidine (1.00 mmol). 215 mg title compound **4b** were obtained as an oil (85% yield).

^1^H NMR (CDCl_3_): *δ*/ppm = 7.55 (m, 4H, ArH), 7.45 (m, 1H, ArH), 7.35 (m, 1H, ArH), 7.13 (m, 2H, ArH), 4.70 (br, 1H, piperidine CH), 3.75 (br, 1H, piperidine CH), 3.01 (br, 1H, piperidine CH), 2.80 (br, 1H, piperidine CH), 1.78 (m, 1H, piperidine C(4)H), 1.63 (m, 2H, piperidine CH), 1.21 (m, 2H, piperidine CH), 0.98 (d, *J* = 6.3 Hz, piperidine CH_3_). ^13^C NMR (CDCl_3_): *δ*/ppm = 21.70, 31.15, 115.69 (d, *J*_HF_ = 16.3 Hz), 125.44 (d, *J*_HF_ = 6.7 Hz), 127.89, 127.74, 127.75, 128.87, 136.50 (d, *J*_HF_ = 3.8 Hz), 137.21, 140.51, 170.05. ESI-MS *m/z*: 298.2 ([M+H]^+^). HR‑MS *m*/*z*: 298.1607 ([M+H]^+^, C_19_H_21_FNO requires 298.1602).

*(4’-Chloro-[1,1’-biphenyl]-3-yl)(4-methylpiperidin-1-yl)methanone [****5b****]*

The reaction was performed according to general procedure D2 using 87 mg **m5** (0.36 mmol) and 53 μL 4‑methylpiperidine (0.43 mmol). 112 mg title compound **5b** were obtained as an oil (quantitative yield).

^1^H NMR (CDCl_3_): *δ*/ppm = 7.60 (m, 2H, ArH), 7.52 (m, 2H, ArH), 7.47, (m, 1H, ArH), 7.42 (m, 1H, ArH), 7.37 (dt, *J* = 7.5, 1.4 Hz, 1H, Ar-H), 4.70 (br, 1H, piperidine CH), 3.75 (br, 1H, piperidine CH), 3.02 (br, 1H, piperidine CH), 2.80 (br, 1H, piperidine CH), 1.78 (br, 1H, piperidine C(4)H), 1.65 (m, 2H, piperidine CH), 1.20 (m, 2H, piperidine CH), 0.99 (d, *J* = 6.4, 3H, piperidine CH_3_). ^13^C NMR (CDCl_3_): *δ*/ppm = 21.78, 31.21, 33.85 (br), 34.80 (br), 42.62 (br), 48.12 (br), 125.43, 125.87. 127.91, 128.41, 128.98, 129.03, 133.83, 137.28, 138.88, 140.32, 170.00. ESI-MS *m/z*: 314.2 ([M(^35^Cl)+H]^+^), 316.2 ([M(^37^Cl)+H]^+^). HR-MS *m*/*z*: 314.1310 ([M+H]^+^, C_19_H_21_ClNO requires 314.1306).

*Azepan-1-yl(4’-chloro-[1,1’-biphenyl]-3-yl)methanone [****5e****]*

The reaction was performed according to general procedure A2 using 259 mg **13e** (0.90 mmol) and 156 mg 4‑chlorophenylboronic acid (0.99 mmol). 237 mg title compound **5e** were obtained after preparative HPLC purification as an off-white solid (83% yield).

Mp: 116-117°C. ^1^H NMR (CDCl_3_): *δ*/ppm = 7.40-7.59 (m, 7H, ArH), 3.71 (m, 2H, azepane CH), 3.41 (m, 2H, azepane CH), 1.87 (m, 2H, azepane CH), 1.65 (m, 6H, azepane CH). ^13^C NMR (CDCl_3_): *δ*/ppm = 26.53, 27.30, 27.90, 29.62, 46.34, 49.84, 125.09, 125.56, 127.59, 128.41, 128.98, 129.04, 133.80, 138.10, 138.93, 140.25, 171.28. ESI-MS *m/z*: 314.2 ([M(^35^Cl)+H]^+^), 316.2 ([M(^37^Cl)+H]^+^). HR-MS *m*/*z*: 314.1309 ([M+H]^+^, C_19_H_21_ClNO requires 314.1306).

*(2’-Chloro-[1,1’-biphenyl]-3-yl)(4-methylpiperidin-1-yl)methanone [****6b****]*

The reaction was performed according to general procedure D2 using 62 mg **m6** (0.26 mmol) and 40 μL 4‑methylpiperidine (0.32 mmol). 68 mg title compound **6b** were obtained as an oil (82% yield).

^1^H NMR (CDCl_3_): *δ*/ppm = 7.46 (m, 5H, ArH), 7.32 (m, 3H, ArH), 4.71 (br, 1H, piperidine CH), 3.84 (br, 1H, piperidine CH), 3.03 (br, 1H, piperidine CH), 2.79 (br, 1H, piperidine CH), 1.75 (m, 1H, piperidine C(4)H), 1.67 (m, 2H, piperidine CH), 1.24 (m, 2H, piperidine CH), 0.99 (d, *J* = 5.4, 3H, piperidine CH_3_). ^13^C NMR (CDCl_3_): *δ*/ppm = 21.78, 31.24, 58.92, 126.28, 127.00, 127.84, 128.41, 128.88, 130.01, 130.41, 132.43, 136.31, 139.35, 139.74, 170.02. The signals of two piperidine CH_2_ are not observed. ESI-MS *m/z*: 314.2 ([M(^35^Cl)+H]^+^), 316.2 ([M(^37^Cl)+H]^+^). HR-MS *m*/*z*: 314.1311 ([M+H]^+^, C_19_H_21_ClNO requires 314.1306).

*(*S*)-1-(2’-Chloro-[1,1’-biphenyl]-3-carbonyl)-pyrrolidine-2-carboxamide [****6f****]*

The reaction was performed according to general procedure D2 using 193 mg **m6** (0.75 mmol) and 171 mg TFA×H-(*S*)-Pro-NH_2_ ( 0.75 mmol). 202 mg title compound **6f** were obtained as an oil containing about 6 % solvent according to NMR analysis (75% yield).

^1^H NMR (CDCl_3_): *δ*/ppm = 7.61 (br, 1H, ArH), 7.53 (m, 4H, ArH), 7.32 (m, 3H, ArH), 6.99 (br, 1H, CONH), 5.46 (br, 1H, CONH’), 4.83 (dd, *J* = 7.5, 4.9 Hz, 1H, Pro αCH), 3.61 (m, 2H, Pro δCH_2_), 2.49 (m, 1H, Pro βCH), 2.08 (m, 2H Pro CH), 1.89 (m, 1H Pro CH). ^13^C NMR (CDCl_3_): *δ*/ppm = 27.14, 50.58, 59.56, 126.48, 127.04, 128.25, 128.36, 129.05, 130.04, 131.32, 131.42, 132.42, 135.90, 139.48, 139.55, 170.92, 179.11.

ESI-MS *m/z*: 314.2 ([M(^35^Cl)+H]^+^), 316.2 ([M(^37^Cl)+H]^+^). HR-MS *m*/*z*: 329.1054 ([M+H]^+^, C_18_H_18_ClN_2_O_2_ requires 329.1051).

*(4’-Hydroxy-[1,1’-biphenyl]-3-yl)(4-methylpiperidin-1-yl)methanone [****7b****]*

172 mg Boc-**7b** (0.44 mmol) were dissolved under argon in 2 mL of a 4:1 dichloromethane/95% aqueous TFA mixture in a round-bottomed flask. After stirring for 2 h at room temperature the mixture was stripped with nitrogen; the residue was taken-up in dichloromethane and stripped with nitrogen 3 more times. The residue was taken-up with diethyl ether and evaporated under reduced pressure; this was repeated 2 times. 125 mg title compound **7b** were obtained as a dry foam (98% yield).

^1^H NMR (CDCl_3_ + 5 % *d*_4_-MeOH): *δ*/ppm = 7.58 (m, 1H, ArH), 7.52 (t, *J* = 1.5 Hz, 1H, ArH), 7.43 (m, 3H, ArH), 7.27, (m, 1H, ArH), 6.90 (m, 2H, ArH), 4.66 (d br, *J* = 11.7 Hz, 1H, piperidine CH), 3.77 (d br, *J* = 12.1 Hz, 1H, piperidine CH), 3.00 (br, 1H, piperidine CH), 2.80 (br, 1H, piperidine CH), 1.79 (m, 1H, piperidine C(4)H), 1.65 (m, 2H, piperidine CH), 1.20, (m, 2H, piperidine CH), 0.98 (d, *J* = 6.2 Hz, piperidine CH_3_). ^13^C NMR (CDCl_3_ + 5 % *d*_4_-MeOH): *δ*/ppm = 21.59, 31.08, 33.77, 34.69, 42.64, 48.26, 115.75, 124.54, 124.76, 127.69, 128.16, 128.81, 131.73, 136.43, 141.40, 156.71, 170.84. ESI-MS *m/z*: 296.2 ([M+H]^+^). HR-MS *m*/*z*: 296.1653 ([M+H]^+^, C_19_H_22_NO_2_ requires 296.1645).

*Azepan-1-yl(4’-hydroxy-[1,1’-biphenyl]-3-yl)methanone [****7e****]*

The reaction was performed according to general strategy B2 using 262 mg **14e** (1.05 mmol) and 175 mg 4‑bromophenol (1.0 mmol). 179 mg title compound were obtained as a white solid after preparative HPLC purification (59% yield).

Mp: 184-185°C. ^1^H NMR (*d_6_*-DMSO): *δ*/ppm = 9.58 (s, 1H, OH), 7.62 (ddd, *J* = 7.8, 1.8, 1.1 Hz, 1H, ArH), 7.51 (m, 3H, ArH), 7.45 (t, *J* = 7.7 Hz, 1H, ArH), 7.23 (ddd, *J* = 7.8, 1.8, 1.1 Hz, 1H, ArH), 6.85 (m, 2H, ArH), 3.57 (t, *J* = 5.8 Hz, 2H, azepane CH), 3.32 (m, 2H, azepane CH) 1.74 (m, 2H, azepane CH), 1.57 (m, 6H, azepane CH). ^13^C NMR (*d_6_*-DMSO): *δ*/ppm = 25.87, 26.81, 27.19, 28.89, 45.35, 49.11, 115.78, 123.54, 124.16, 126.35, 127.84, 128.89, 130.11, 137.98, 140.19, 157.38, 170.17. ESI-MS *m/z*: 296.2 ([M+H]^+^). HR-MS *m*/*z*: 296.1651 ([M+H]^+^, C_19_H_22_NO_2_ requires 296.1645).

*(3’-Hydroxy-[1,1’-biphenyl]-3-yl)(4-methylpiperidin-1-yl)methanone [****8b****]*

The reaction was performed according to general strategy D2 using 188 mg **m8** (0.94 mmol) and 130 μL 4-methylpiperidine (1.05 mmol). 295 mg title compound **8b** were obtained as an oil containing about 11% solvent according to NMR analysis (quantitative yield).

^1^H NMR (CDCl_3_): *δ*/ppm = 7.51 (t, *J* = 1.2 Hz, 1H, ArH), 7.35 (dt, *J* = 7.5, 1.3 Hz, 1H, ArH), 7.47 (dt, *J* = 7.0, 1.8 Hz, 1H, ArH), 7.35 (m, 2H, ArH), 7.23 (m, 1H, ArH), 7.06 (br, 1H, OH), 7.01 (dt, *J* = 7.6, 1.3 Hz), 6.81 (m, 2H, ArH), 4.73 (m, 1H, piperidine CH), 3.75 (m, 1H, piperidine CH), 3.02 (m, 1H, piperidine CH), 2.82 (m, 1H, piperidine CH), 1.79 (br, 1H, piperidine C(4)H), 1.64 (m, 2H, piperidine CH), 1.22 (m, 2H, piperidine CH), 0.99 (d, *J* = 6.4 Hz, 3H, CH_3_). ^13^C NMR (CDCl_3_): *δ*/ppm = 21.70, 31.15, 33.86 (br), 34.76 (br), 42.74 (br), 48.25 (br), 114.16, 114.95, 118.90, 125.30, 125.49, 128.23, 128.87, 129.84, 136.42, 141.36, 141.65, 156.79, 170.69. ESI-MS *m/z*: 296.2 ([M+H]^+^). HR-MS *m*/*z*: 296.1651 ([M+H]^+^, C_19_H_22_NO_2_ requires 296.1645).

*3’-(4-methylpiperidine-1-carbonyl)-[1,1’-biphenyl]-4-carboxamide [****9b****]*

The reaction was performed according to general strategy D2 using 220 mg **m9** (0.89 mmol) and 132 μL 4-methylpiperidine (1.07 mmol). 188 mg title compound **9b** were obtained as a white solid (64% yield).

Mp: 188-190°C. ^1^H NMR (CDCl_3_): *δ*/ppm = 7.90 (m, 2H, ArH), 7.64 (m, 4H, ArH), 7.49 (m 1H, ArH), 6.35 (br, 1H, CONH), 5.85 (br, 1H, CONH’), 4.70 (br, 1H, piperidine CH), 3.75 (br, 1H, piperidine CH), 3.02 (br, 1H, piperidine CH), 2.80 (br, 1H, piperidine CH), 1.79 (br, 1H piperidine C(4)H), 1.64 (m, 2H, piperidine CH), 1.24 (m, 2H, piperidine CH), 0.99 (d, *J* = 6.4 Hz, 3H, CH_3_). ^13^C NMR (CDCl_3_): *δ*/ppm = 21.74, 31.19, 33.82 (br), 34.82 (br), 42.60 (br), 48.17 (br), 125.66, 126.30, 127.23, 128.07, 128.17, 129.05, 132.39, 137.29, 140.30, 143.87, 168.95, 169.96. ESI-MS *m/z*: 323.2 ([M+H]^+^). HR-MS *m*/*z*: 323.1758 ([M+H]^+^, C_20_H_23_N_2_O_2_ requires 323.1754).

*3’-(4-methylpiperidine-1-carbonyl)-[1,1’-biphenyl]-3-carboxamide [****10b****]*

The reaction was performed according to general strategy D2 using 211 mg **m10** (0.87 mmol) and 128 μL 4-methylpiperidine (1.04 mmol). 278 mg title compound **10b** were obtained as a dry foam containing about 5% solvent according to NMR analysis (93% yield).

^1^H NMR (CDCl_3_): *δ*/ppm = 8.05 (t, *J* = 1.8 Hz, ArH), 7.80 (dt, *J* = 7.6, 1.3 Hz, 1H, ArH), 7.74 (dt, *J* = 8.1, 1.4 Hz, 1H, ArH), 7.64 (m, 2H, ArH), 7.49 (m, 2H, ArH), 7.37 (dt, *J* = 7.5, 1.3 Hz, 1H, ArH), 6.40 (br, 1H, CONH), 5.84 (br, 1H, CONH’), 4.70 (br, 1H, piperidine CH), 3.75 (br, 1H, piperidine CH), 3.02 (m, 1H, piperidine CH), 2.80 (m, 1H, piperidine CH), 1.78 (br, 1H, piperidine C(4)H), 1.67 (m, 2H, piperidine CH), 1.23 (m, 2H, piperidine CH), 0.98 (d, *J* = 6.4 Hz, 3H, CH_3_). ^13^C NMR (CDCl_3_): *δ*/ppm = 21.74, 31.19, 33.87 (br), 34.69 (br), 42.57 (br) 48.26 (br), 125.65, 125.99, 126.30, 126.49, 128.13, 129.01, 129.18, 130.58, 134.07. 137.28, 140.50, 140.91, 169.14, 170.02. ESI-MS *m/z*: 323.2 ([M+H]^+^). HR-MS *m*/*z*: 323.1757 ([M+H]^+^, C_20_H_23_N_2_O_2_ requires 323.1754).

*Azepan-1-yl(3’,4’-dimethyl-[1,1’-biphenyl]-3-yl)methanone [****11e****]*

The reaction was performed according to general strategy B2 262 mg **14e** (1.05 mmol) and 136 μL 4-bromo-1,2-dimethylbenzene (1.0 mmol). 192 mg title compound **11e** were obtained after preparative HPLC purification as an oil (63% yield).

^1^H NMR (CDCl_3_): *δ*/ppm = 7.60 (m, 2H, ArH), 7.43 (m, 1H, ArH), 7.39 (br, 1H, ArH), 7.33 (m, 2H, ArH), 7.21 (br, 1H, ArH), 3.71 (t, *J* = 5.8 Hz, 2H, azepane CH), 3.41 (m, 2H, azepane CH), 2.34 (s, 3H, ^3’^CH_3_), 2.32 (s, 3H, ^4’^CH_3_), 1.87 (m, 2H, azepane CH), 1.62 (m, 6H, azepane CH). ^13^C NMR (CDCl_3_): *δ*/ppm = 19.48, 19.94, 26.53, 27.33, 27.92, 29.61, 46.56, 49.83, 124.48, 124.89, 125.01, 127.57, 128.42, 128.75, 130.16, 136.14, 137.06, 137.86, 138.07, 141.45, 171.62.

ESI-MS *m/z*: 308.3 ([M+H]^+^). HR-MS *m*/*z*: 308.2013 ([M+H]^+^, C_21_H_26_NO requires 308.2009).

*[1,1’-Biphenyl]-3-yl(azepan-1-yl)methanone [****12e****]*

The reaction was performed according to strategy B2 using 262 mg **14e** (1.05 mmol) and 106 μL bromobenzene (1.0 mmol). 250 mg title compound **12e** were obtained as a white solid (86% yield).

Mp: 52-53°C. ^1^H NMR (CDCl_3_): *δ*/ppm = 7.61 (m, 4H, ArH), 7.46 (m, 3H, ArH), 7.37 (m, 2H, ArH), 3.71 (t, *J* = 5.8 Hz, 2H, azepane CH), 3.42 (m, 2H, azepane CH), 1.83 (m, 2H, azepane CH), 1.64 (m, 6H, azepane CH). ^13^C NMR (CDCl_3_): *δ*/ppm = 26.33, 27.32, 27.90, 29.62, 46.38, 49.85, 125.21, 125.28, 127.17, 127.64, 128.98, 137.93, 140.19, 141.44, 171.51. ESI-MS *m/z*: 280.2 ([M+H]^+^). HR-MS *m*/*z*: 280.1702 ([M+H]^+^, C_19_H_22_NO requires 280.1696).

References and notes

[1] M. Venkatraj, J. Messagie, J. Joossens, A.M. Lambeir, A. Haemers, P. Van der Veken, K. Augustyns, Synthesis and evaluation of non-basic inhibitors of urokinase-type plasminogen activator (uPA), Bioorganic & medicinal chemistry, 20 (2012) 1557-1568.

[2] D.N. Korolev, N.A. Bumagin, Pd-EDTA as an eficient catalyst for Suzuki-Miyaura reactions in water, Tetrahedron Lett, 46 (2005) 5751-5754.

[3] M.J. Burk, J.R. Lee, J.P. Martinez, A versatile tandem catalysis procedure for the preparation of novel amino acids and peptides, J Am Chem Soc, 116 (1994) 10847 10848.

1. Most of the spectrum consists of two series of broad signals of equal intensity (conformers). [↑](#footnote-ref-2)
